# Time-resolved growth of diverse human-associated *Akkermansia* on human milk oligosaccharides

**DOI:** 10.1128/spectrum.02071-25

**Published:** 2026-01-27

**Authors:** Ashwana D. Fricker, Abigail L. Barnes, Arno Henzi, Luis E. Duran, Gilberto E. Flores

**Affiliations:** 1 Department of Biology, California State University, Northridge, California, USA; 2 Biology Department, Adelphi University 3479, Garden City, New York, USA; Temasek Life Sciences Laboratory, Singapore, Singapore

**Keywords:** *Akkermansia*, human milk oligosaccharides, short-chain fatty acids

## Abstract

The infant gut microbiota is strongly influenced by human milk oligosaccharides (HMOs), a set of glycans that comprise a large constituent of milk and reach the large intestine intact. During growth on HMOs, bacteria produce beneficial metabolites, including short-chain fatty acids (SCFAs), that are important for host health. Select gut microorganisms have unique sets of enzymes capable of catabolizing distinct HMOs, leading to host-specific differences in glycan access and ultimately differences in SCFA production. Here, we cultivated three species of human-associated *Akkermansia,* an early-life commensal that is correlated with a healthy metabolic status in adults, on five individual HMOs in two different media backgrounds. Analysis of growth rates, growth yield, metabolic output, and individual HMO consumption through time revealed differences across species that were influenced by growth media. Most notably, *Akkermansia biwaensis* CSUN-19 has robust growth in both media backgrounds paired with nearly complete degradation of all HMOs. Across all conditions, overall SCFA production was generally commensurate with growth, but most strikingly, *Akkermansia muciniphila* MucT and *A. biwaensis* CSUN-19 produced succinate only when grown in the presence of N-acetyl glucosamine, but not with mucin. The third organism tested, *Akkermansia massiliensis* CSUN-17, had weaker growth, lower degradation of HMOs, but higher production of propionate in media containing N-acetyl glucosamine. Interactions between *Akkermansia* and HMOs can influence colonization of other early life commensals, potentially influencing health outcomes throughout life. This study highlights the importance of characterizing the growth of individual *Akkermansia* species on distinct HMO leading to fermentation into organic acids.

**IMPORTANCE:**

*Akkermansia* is a widely distributed bacterial genus found in the healthy human gut that is capable of degrading host-produced glycans, including human milk oligosaccharides (HMOs). Previous endpoint experiments demonstrated varying degradation efficiencies across *Akkermansia* species, with *A. biwaensis* displaying enhanced growth on multiple HMOs. However, the temporal dynamics and growth preferences when offered substrate choice across the lineage are unknown. Here, we characterized the temporal growth dynamics, HMO catabolism, and metabolic output of three *Akkermansia* species across five HMOs and two media backgrounds. Specifically, we demonstrate that one species, *A. biwaensis* CSUN-19, has robust growth independent of media background with nearly complete degradation of all HMOs tested. Overall, the species-, HMO-, and media-specific response of *Akkermansia* may impact the colonization success of each species, ultimately influencing host–microbe and microbe–microbe interactions in the developing infant gut microbiome.

## INTRODUCTION

Human milk is a unique and highly complex fluid that provides all the necessary nutritional requirements for the growth of a developing infant (1, 2). In addition to nutrition, milk provides important bioactive components, including sugars, lipids, enzymes, immunoglobulins, hormones, vitamins, and minerals, that provide a range of benefits to the growing infant (1). Sugars in human milk are highly abundant and can be found as lactose, free human milk oligosaccharides (HMOs), or conjugated to proteins or lipids (2). HMOs are the third most abundant component of human milk (5–15 g/L) following lactose and lipids (3, 4). HMOs are composed of a pool of over 200 intricate individual sugars, but only five monosaccharides make up all HMO structures. These are glucose, galactose, N-acetylglucosamine (GlcNAc), fucose, and N-acetylneuraminic acid (NeuAc, sialic acid). Structurally, HMOs consist of a lactose core with the aforementioned monomers linked to this core, resulting in structures that are linear or branched and can be complex or relatively simple (5). Simple yet abundant HMOs include 2′-fucosyllactose (2′-FL), 3-fucosyllactose (3-FL), lacto-N-tetraose (LNT), lacto-N-neotetraose (LNnT), and 6’-sialyllactose (6′-SL) (2, 4). Interestingly, HMOs are not a nutrient source for the developing infant; instead, these plentiful sugars influence the infant gut microbiota assembly by inhibiting pathogen colonization and enriching for beneficial bacteria (6–10).

The infant microbiota is typically dominated by HMO-catabolizing bacteria, most notably from the *Bifidobacterium* genus (reviewed in reference 11). While *Bifidobacterium* is typically dominant in these communities, other bacteria with HMO-degrading capabilities are also commonly present (11). Another genus of organisms present and capable of growing in early life is *Akkermansia*, a lineage of mucin-degrading specialists that is largely regarded as beneficial bacteria in adults (12, 13). All known species of *Akkermansia* have a large repertoire of glycoside hydrolase (GHs) enzymes that are involved in mucin catabolism. However, given the structural and compositional similarity between mucin and HMOs (4, 14), GHs implicated in mucin catabolism in *Akkermansia* are also likely capable of breaking down HMOs. Importantly, individual species of *Akkermansia* have different sets of GHs, suggesting differences in catabolic capacity across species (15, 16). While the original description of growth on HMOs used *Akkermansia muciniphila* (17), our lab has recently demonstrated endpoint growth differences across *Akkermansia* species when grown on individual HMOs and mucin (15). These differences in growth are likely due to the presence and regulation of individual enzymes (16).

Given the endpoint differences in growth and HMO consumption across *Akkermansia* species in a mucin background, we wanted to examine the temporal dynamics of HMO degradation in different media backgrounds. We hypothesized that each species would consume HMOs at different rates and produce different concentrations of short-chain fatty acids (SCFA) as fermentation products. We first determined the growth of representative isolates from three named human-associated *Akkermansia* species on five individual HMOs. Subsequently, we selected six time points throughout growth for SCFA and catabolite quantification to identify when individual sugars are digested. Furthermore, to disentangle the response to HMOs from the other sugars present in mucin, synthetic media containing GlcNAc instead of mucin were tested in parallel. From this, we demonstrate differences in growth on individual HMOs, of which one species, *Akkermansia biwaensis*, has a robust growth response on multiple HMOs. This robust growth on multiple HMOs across two media demonstrates the potential importance of *A. biwaensis* in the infant microbiota.

## MATERIALS AND METHODS

### Growth on individual HMOs

*Akkermansia* sp. were grown on basal tryptone threonine medium (BTTM, based on reference 18) in the presence of 10 mM GlcNAc or 0.5% mucin and HMOs, conditions for which we have previously demonstrated robust growth (15, 16). Prior to inoculation, cultures were subcultured twice from glycerol freezer stocks at a 10% and 2% inoculum, respectively, in 5 mL BTTM with 0.4% vol/vol soluble type III porcine gastric mucin (Sigma-Aldrich) prepared as described previously with overnight growth at 37°C (15, 19). Prior to seeding into the experiment, each species was normalized to an OD_600nm_ of 0.5 with carbon-free basal medium and subsequently inoculated 1:10 into BTTM containing the respective carbon sources. Each tube of BTTM contained a final concentration of 4 mM HMOs (2′-FL, 3-FL, 6′-SL, LNT, LNnT) or lactose and 0.4% mucin or 8mM GlcNAc (Table S1). HMOs were selected based on structural simplicity, abundance in natural breastmilk, availability, and previous endpoint studies with these same *Akkermansia* strains (15, 20). During media preparation, HMOs, lactose, milliQ water, mucin, and GlcNAc were filter sterilized (UNIFLO 13 mm 0.2 mM PES Filter Media, Whatman). All cultures were prepared and maintained in a Vinyl Anaerobic Chamber (Coy Laboratory Products, Incorporated, Grass Lake, MI) with a headspace of 80% N_2_, 15% CO_2_, and 5% H_2_.

To monitor growth, OD_600nm_ was read hourly using a SpectroSTAR nano spectrophotometer (BMG Labtech, Germany) in clear-bottom 96-well plates (Falcon, USA) containing 200 mL of the respective cultures. After inoculation, plates were sealed with a Breathe-easy film (Diversified Biotech) to allow for gas exchange, and plates were maintained static at 37°C except for a 10 s orbital shake prior to each hourly read. All experimental cultures were grown in triplicate, and experiments were repeated four times. Values from the SpectroSTAR nano spectrophotometer plate reader were converted to true OD_600nm_ using a linear conversion. To normalize readings, the initial read (zero time point) was subtracted from all subsequent values. Although rare, individual wells with abnormal growth curves were removed from further calculations.

For experiments involving metabolite detection and tracking of HMO degradation, after preparing cultures, 0.5 mL aliquots were distributed into 2 mL microfuge tubes and maintained static separately at 37°C, anaerobically. At the indicated time points, each aliquot was centrifuged at 15,000 rcf for 5 min, and supernatants were stored at −20°C until further processing (below).

Doubling time and hour at maximum slope was calculated using a rolling slope method. Slopes were calculated across every 5-h window, and the maximum slope during exponential growth was used to determine the doubling time by calculating the ln(2)/slope using the *zoo* package (v.1.8-12) in R. Hypothesis testing was performed using Kruskal-Wallis, followed by a post hoc Dunn’s test using the *rstatix* package (0.7.2). R scripts are provided as Files S1 to S3.

### Metabolite and HMO profiling

To determine levels of SCFAs (acetate, propionate, and succinate), sugars (lactose, GlcNAc, fucose, sialic acid), and HMOs (2′-FL, 3-FL, 6′-SL, LNT, LNnT) samples were analyzed using high-performance liquid chromatography (HPLC). Prior to analysis, samples maintained at −20°C were filtered (Millex, 13 mm, 0.2 uM PTFE Membrane, Millipore) to prevent clogging of the column. Filtered supernatants were measured using an Agilent 1260 HPLC system (Agilent, USA) equipped with a Varian Metacarb 67H column (300 by 6.5 mm) maintained at 45°C with 5 mM sulfuric acid at a flow rate of 0.8 mL/min. A refractive index detector at 35°C was used to measure glycans and metabolites. Concentrations were determined by comparing the area under the curve to six-point standards for each compound of interest; a chemical list of all standards is provided in Table S2. Graphs were generated using the *ggplot2* package (v.3.5.1), and significant differences between samples were calculated using the *rstatix* package in R v.4.2.3.

### Vitamin B12 bioassay

To determine if vitamin B12 was present in the mucin used as a growth substrate, we used the *Lactobacillus leichmannii* ATCC 7830 bioassay. For this bioassay, the original protocol was modified and described in previous work (21, 22). Briefly, sterile vitamin B12 assay medium (BD Difco) with standard concentrations (0 to 2.5 ng/mL) of cyanocobalamin (Sigma-Aldrich) or different batches of mucin to a final concentration of 0.05% (note: this represents a 10× lower concentration than in media used to grow *Akkermansia*). Cyanocobalamin and mucin were filter sterilized prior to addition to cooled assay media (UNIFLO 13 mm 0.2 mM PES Filter Media, Whatman). After media preparation, overnight cultures of *L. leichmannii* grown in MRS at 37°C in 5% CO_2_ were pelleted at 15,000 rcf for 3 min and washed 3× with sterile Milli-Q water. Pellets were resuspended in sterile water and incubated at 4°C for 1 h. This culture was diluted 1:10 with sterile water before inoculating 1:100 into 1 mL vitamin B12 assay medium (BD Difco) in a 48-well plate. Cultures were incubated for 20 h at 37°C and 5% CO_2_. Growth was measured with a Spectromax M5 (Molecular Devices, USA) at OD_600nm_. Values from the Spectromax plate reader were converted to true OD_600nm_ using a linear conversion.

## RESULTS

### Growth on HMO is species and media dependent

**Fig 1:**
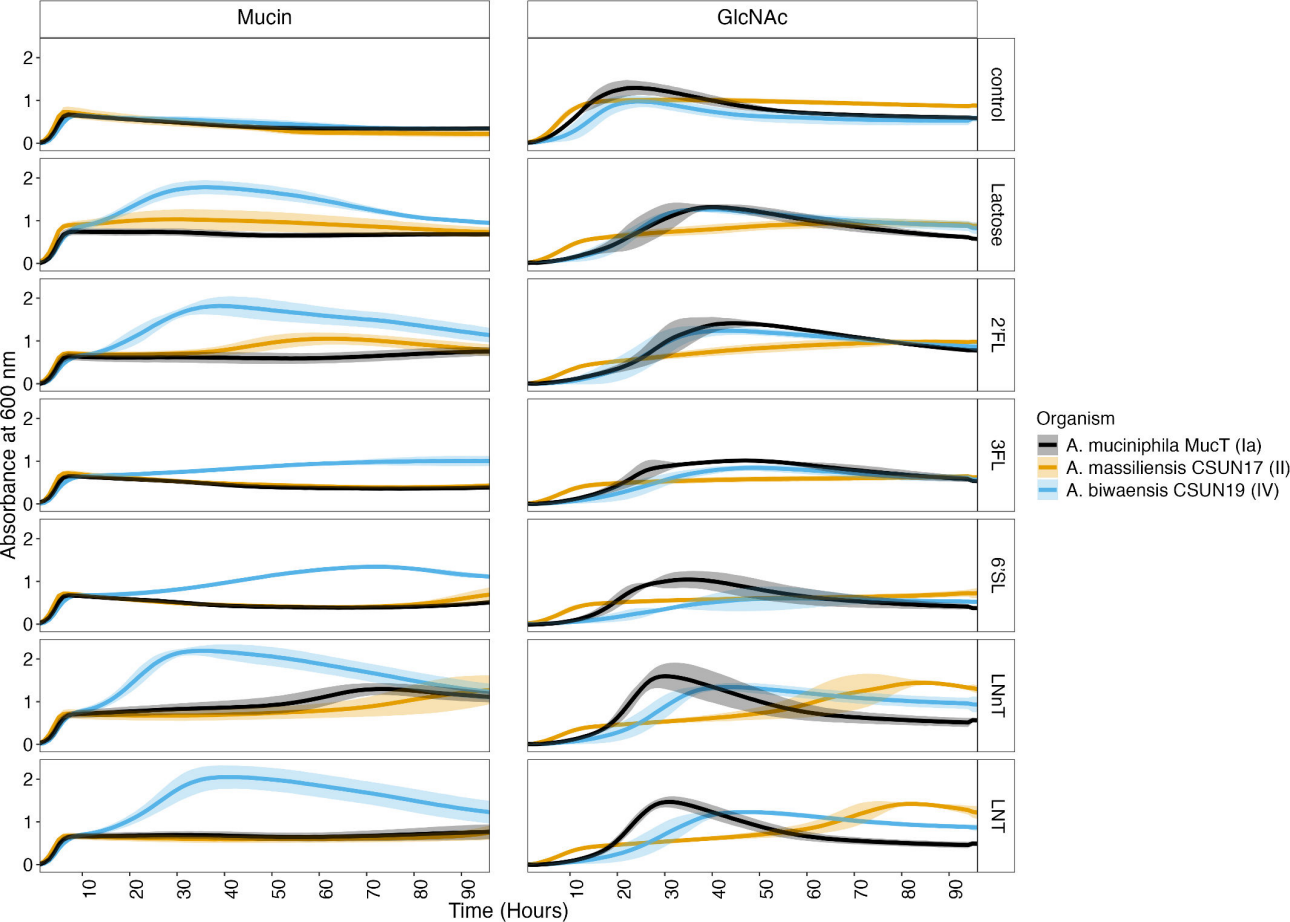
*Akkermansia* species demonstrate different growth responses to individual HMOs through time in a media-dependent manner. OD_600_ was measured hourly for 96 h for three species across five HMOs in the presence of either mucin or GlcNAc. To normalize OD, the initial time point (t = 0 h) was subtracted from all subsequent reads. Values represent the average of three to four biological replicates, each performed in triplicate. Error bars represent the standard deviation of biological replicates as calculated in R.

To identify differences in growth patterns on HMOs across species of human-associated *Akkermansia*, we first cultivated a representative of each named species on HMOs in the presence of mucin. A time-resolved approach revealed differences in species-level growth that were HMO-dependent (Fig. 1).

#### Species vs species

In the mucin medium lacking HMOs, all three representatives had equal growth, reaching equal OD_600_ maxima within 6 h with a slight delay in *A. biwaensis* CSUN-19 (Fig. S1A and E). However, across all five HMOs tested in a mucin background, *A. biwaensis* CSUN-19 had the most robust growth, with significantly higher OD_600_ maxima compared to the other species tested (*P* < 0.01, Fig. S1A and D).

To eliminate the complexity of mucin potentially influencing growth, the same experiment was repeated in the absence of mucin. Instead, GlcNAc was included in the medium to alleviate the auxotrophy for this substrate. As a control, glucose was provided as a carbon and energy source, which equally supported the growth of all species (Fig. 1; Fig. S1A and D). In the presence of GlcNAc, the influence of the HMOs varied drastically across species (Fig. 1). For example, both *A. muciniphila* Muc^T^ and *A. biwaensis* CSUN-19 had similar growth patterns on lactose, 2′-FL, and 3-FL, whereas *Akkermansia massiliensis* CSUN-17 seemed to have an initial early growth phase with a continued gradual increase in OD_600_ over the remaining time points on these same three sugars. Despite the early and continued growth of these three sugars, *A. massiliensis* CSUN-17 had the lowest OD_600_ maximum (Fig. S1A). Comparatively, each species had different growth patterns on 6′-SL; on this HMO in the presence of GlcNAc, *A. muciniphila* Muc^T^ grew abundantly after a ~12 h lag and had the highest maximum OD_600_, whereas both *A. massiliensis* CSUN-17 and *A. biwaensis* CSUN-19 had gradual increases in OD_600_. Across both LNT and LNnT, the patterns of growth appeared similar, with *A. massiliensis* CSUN-17 demonstrating biphasic growth, and *A. muciniphila* Muc^T^ and *A. biwaensis* CSUN-19 growing relatively quickly after a long lag. Notably, all organisms had the same final growth yield on LNnT and similar growth on LNT in a GlcNAc, but not mucin, background (Fig. S1A).

All isolates had a slight decrease in OD_600_ during late stages of growth on mucin alone, which did not occur in the presence of HMOs or lactose. This led to significant differences in the growth with additional sugars compared to mucin alone at both 24 and 48 h (*P* < 0.05, Fig. S2), where the general trends are in line with previous work (15). Curiously, during growth on GlcNAc across multiple HMOs, *A. muciniphila* Muc^T^ had a marked decrease in OD_600_ after peak growth, although this effect was most pronounced in media with LNT or LNnT (Fig. 1).

**Fig 2:**
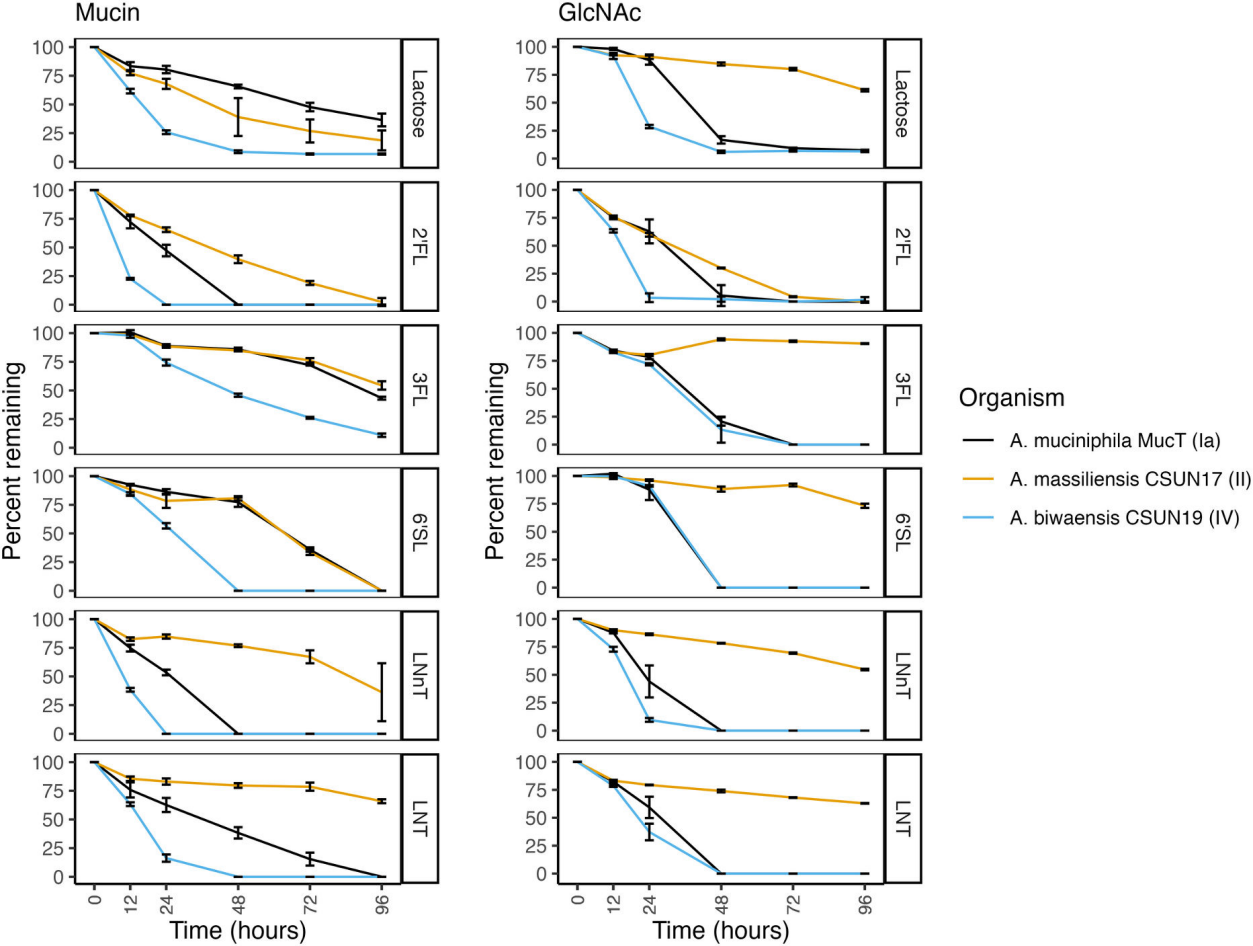
HMO degradation rates by *Akkermansia* are species- and media-dependent. Percent decrease as compared to time zero of each HMO was determined at five time points by HPLC and during growth on either mucin (left) or GlcNAc (right). Error bars represent the standard deviation of three technical replicates.

#### Media vs media

The influence of the media was different across species; for example, *A. muciniphila* Muc^T^ had generally better growth on GlcNAc, *A. massiliensis* CSUN-17 had approximately equal growth on both media, and *A. biwaensis* CSUN-19 grew better overall in a mucin background (Fig. 1). This trend was most noticeable when comparing the maximum OD_600_ and the time at which this maximum was reached (Fig. S1B and E).

#### HMO vs HMO

Within each species, the ability to grow on different HMOs was compared. In the mucin background, *A. massiliensis* CSUN-17 had similar OD_600_ maxima when presented with different HMOs, whereas in a GlcNAc background, there was much greater variability across HMOs (Fig. S1C and F). All three species had higher OD_600_ maxima on LNnT and LNT as compared to the HMO that supported the least amount of growth, 3-FL (Fig. S1C).

To quantify diauxic growth patterns, the maximum doubling time and the midpoint hour at which the slope was calculated were compared for the first and second log phases, when present. Strikingly, across all species and HMOs in a mucin background, the doubling time during the first log phase was similar (Fig. S3). However, there were large differences in the doubling time during the second log phase that was HMO-dependent. This suggests early growth on mucin and subsequent growth on HMO. However, in a GlcNAc background, only *A. massiliensis* CSUN-17 had diauxic growth or continued increases in growth across multiple HMOs occurring after 48 h of incubation (Fig. 1). These doubling times for CSUN-17 suggest early growth on GlcNAc and subsequent use of HMO and point to potential preference for LNT and LNnT.

**Fig 3:**
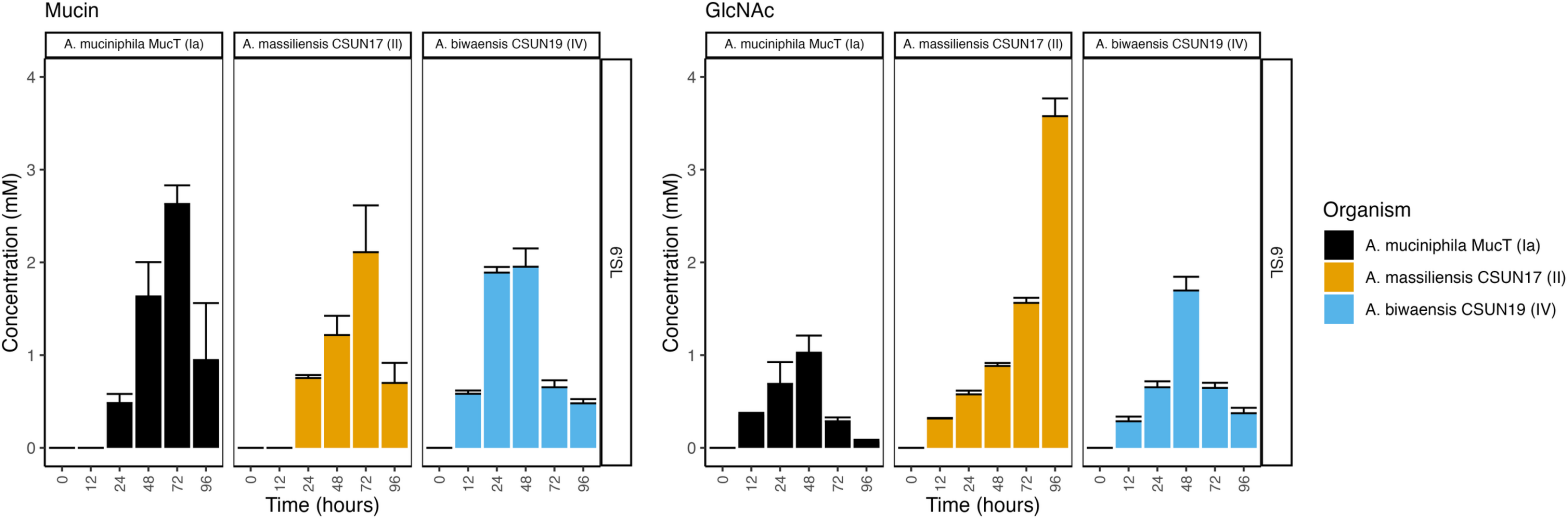
Free sialic acid concentrations peak at 48–72 h, followed by a decrease as *Akkermansia* deconstructs 6′-siallylactose. Concentrations of sialic acid were determined at six time points by HPLC during growth on either mucin (left) or GlcNAc (right). Error bars represent the standard deviation of three technical replicates.

### Rates of HMO degradation are species and HMO dependent

To determine whether rates of HMO degradation matched growth patterns, culture supernatants were analyzed using HPLC before incubation, at 12 h, and subsequently every 24 h throughout growth. In a mucin background, the organism that had the most growth across all HMOs, *A. biwaensis* CSUN-19, also degraded each of the HMOs faster than the other species (Fig. 2). For example, 2′-FL and LNnT were completely digested by 24 h. Similarly, *A. muciniphila*, Muc^T^, catabolized all the 2′-FL and LNnT within 48 h. Surprisingly, in the mucin background, all three organisms catabolized 6′-SL, despite weak growth on this HMO by both *A. muciniphila* Muc^T^ and *A. massiliensis* CSUN-17.

When grown with GlcNAc, the patterns of decreasing HMO concentrations were different from those during growth on mucin. For example, both *A. muciniphila* Muc^T^ and *A. biwaensis* CSUN-19 had similar patterns of 3-FL and 6′-SL on GlcNAc media, whereas in a mucin background, *A. muciniphila* Muc^T^ more similarly resembled *A. massiliensis* CSUN-17 on these HMOs. Across all HMOs except 2′-FL, *A. massiliensis* CSUN-17 did not appear to robustly degrade any in the absence of mucin, suggesting this organism may have primarily used GlcNAc as a carbon and energy source. To address this, levels of GlcNAc were measured using HPLC (Fig. S4). There was a large drop in the levels of GlcNAc within the first 12 h, whereas *A. muciniphila* Muc^T^ and *A. biwaensis* CSUN-19 appeared to use GlcNAc within the 12–24 h window, suggesting a need to acclimate to the absence of mucin. In addition, while all the GlcNAc was consumed by both *A. muciniphila* Muc^T^ and *A. biwaensis* CSUN-19 within 48 h across all HMOs (with the singular exception of *A. biwaensis* CSUN-19 grown on LNT), there was residual GlcNAc in all *A. massiliensis* CSUN-17 cultures by the end of the experiment (96 h).

**Fig 4:**
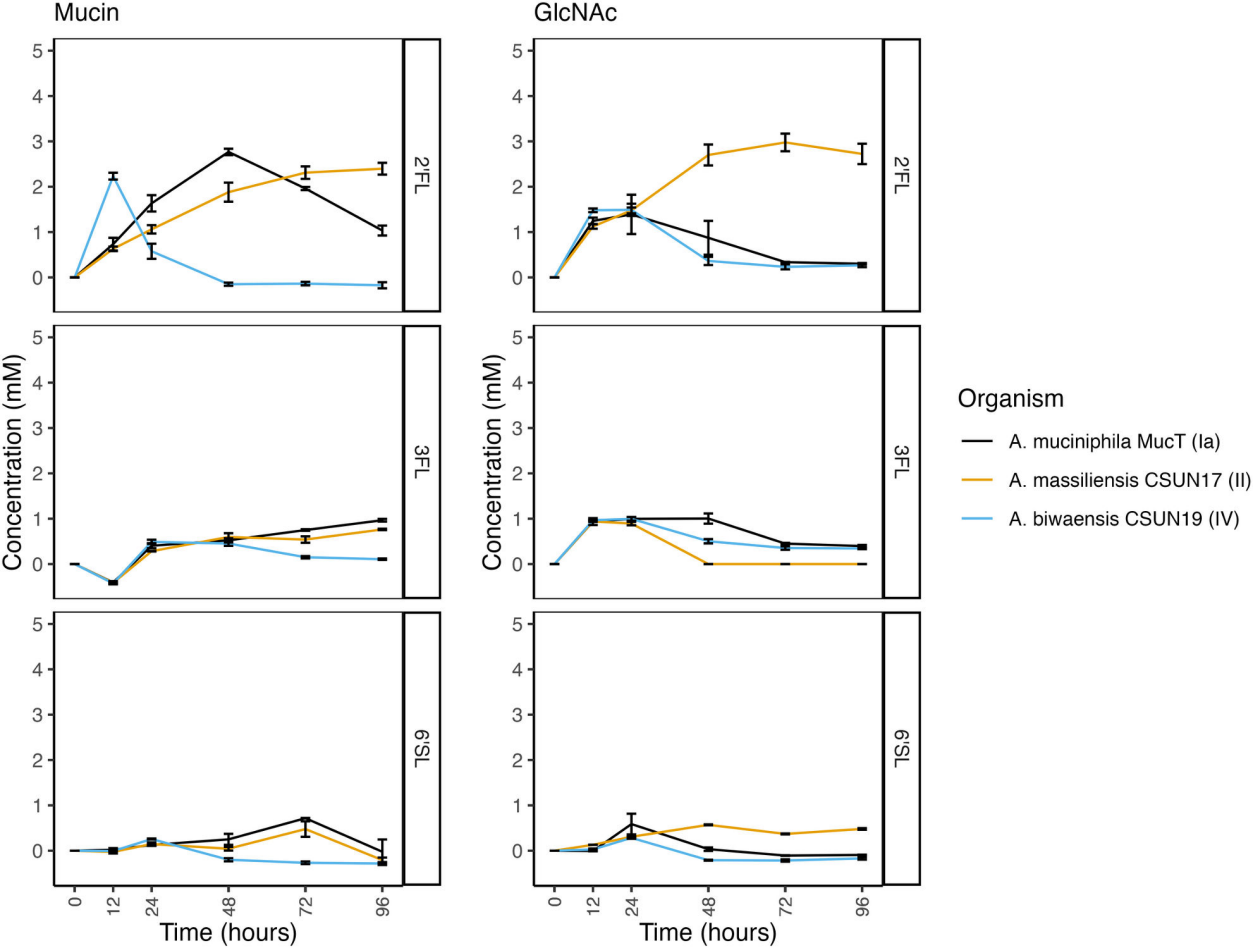
Increase in lactose is species-dependent. Concentrations of lactose were determined at six time points by HPLC during growth on either mucin (left) or GlcNAc (right). Error bars represent the standard deviation of three technical replicates.

### Byproducts of HMO breakdown suggest metabolism of intermediates

In hand with the breakdown of HMOs, levels of sugar breakdown products were determined using HPLC. Trace amounts of fucose (less than 1 mM) were detected in most cultures grown on fucosyllated HMOs; however, the levels were close to the detection limit (data not shown). Perhaps surprisingly, although the concentration of NeuAc increased over the first 48–72 h in all three organisms across both media backgrounds supplemented with 6′-SL, the concentration decreased at later growth stages, with the exception of *A. massiliensis* CSUN-17 in a GlcNAc background (Fig. 3).

Given that lactose forms the backbone of all HMOs, we also measured free lactose in the culture medium for all three species across both media backgrounds (Fig. 4). However, lactose peaks overlapped with unidentified di- or tri-saccharide peaks in media containing LNT and LNnT, precluding lactose measurements for those HMOs. Lactose was transiently detected in nearly all cultures of *A. biwaensis* CSUN-19, likely due to quick consumption. In cultures of *A. muciniphila* Muc^T^ grown on 2′-FL, levels of lactose peaked at 48 h in a mucin background and at 24 h in a GlcNAc background and then slowly diminished throughout the rest of the experiment. For this organism on 3-FL and 6′-SL, lactose concentrations never exceeded 1 mM. All cultures of *A. massiliensis* CSUN-17 appeared to let lactose accumulate in the culture medium throughout the course of the experiment during growth on 2′-FL in both media backgrounds.

### *A. biwaensis* produces greater amounts of SCFAs

**Fig 5:**
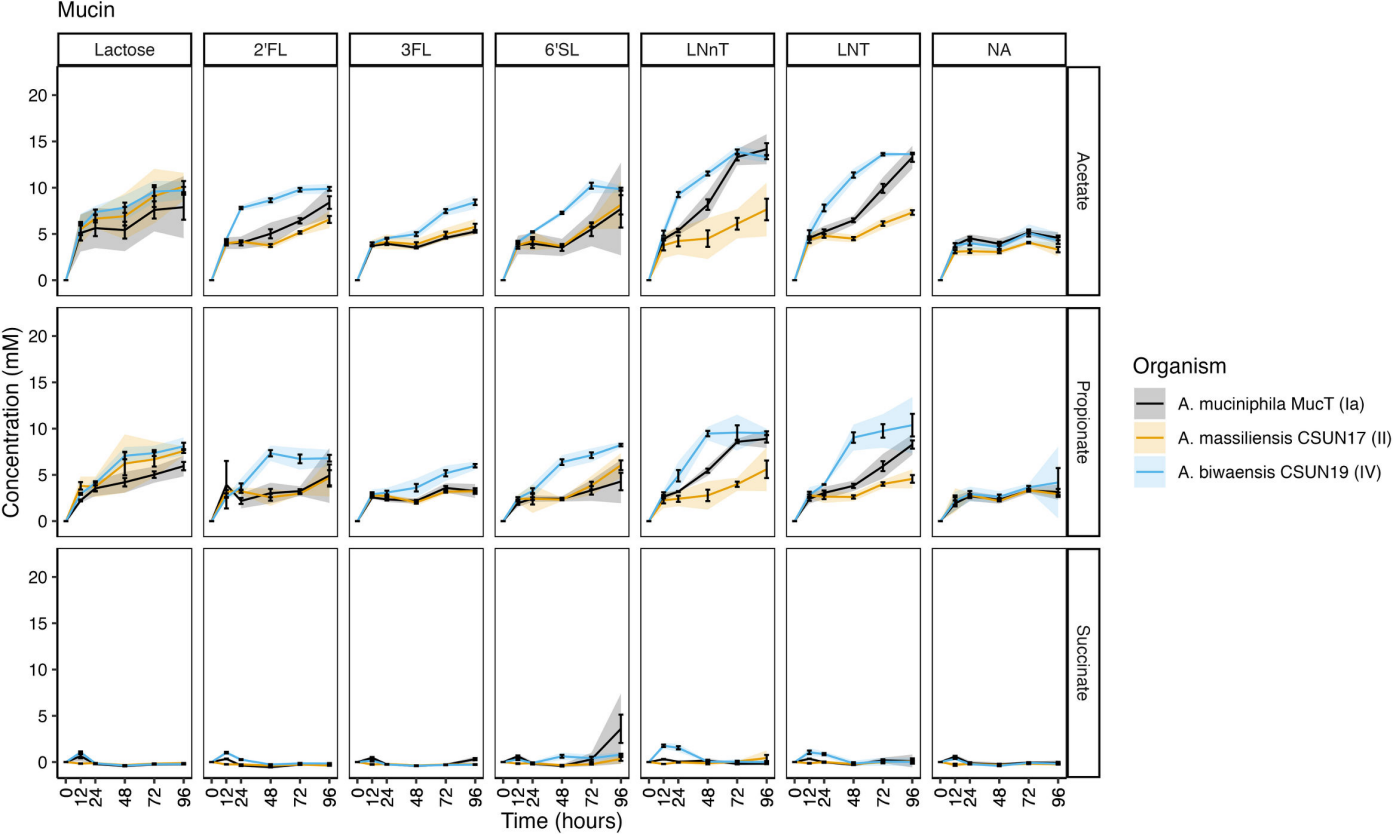
Short-chain fatty acids are produced continuously throughout growth on mucin and HMOs. Concentration of acetate, succinate, and propionate was determined at six time points by HPLC. Error bars represent standard deviation, and shading represents 95% confidence intervals of three technical replicates.

To identify whether individual HMOs led to differences in metabolic output, SCFAs were measured from the same six time points (0, 12, 24, 48, 72, and 96 h) of each isolate. For all three organisms under mucin-only conditions, approximately 5 mM acetate and 3 mM propionate were produced, levels which fluctuated marginally after 12 h (Fig. 5). Similarly, under growth with lactose and mucin, both *A. massiliensis* CSUN-17 and *A. biwaensis* CSUN-19 produced similar levels of acetate and propionate, whereas *A. muciniphila* Muc^T^ produced significantly less propionate (*P* < 0.05 at 96 h). Across all HMOs in a mucin background, *A. biwaensis* CSUN-19 produced the most SCFAs. Notably, despite ample production of acetate and propionate during growth on lactose, *A. massiliensis* CSUN-17 demonstrated equal or weaker production of these SCFAs as compared to *A. muciniphila* Muc^T^ when grown with HMO. Interestingly, both *A. biwaensis* CSUN-19 and *A. muciniphila* Muc^T^ had small amounts of succinate production, which built up slightly in the culture medium before disappearing.

**Fig 6:**
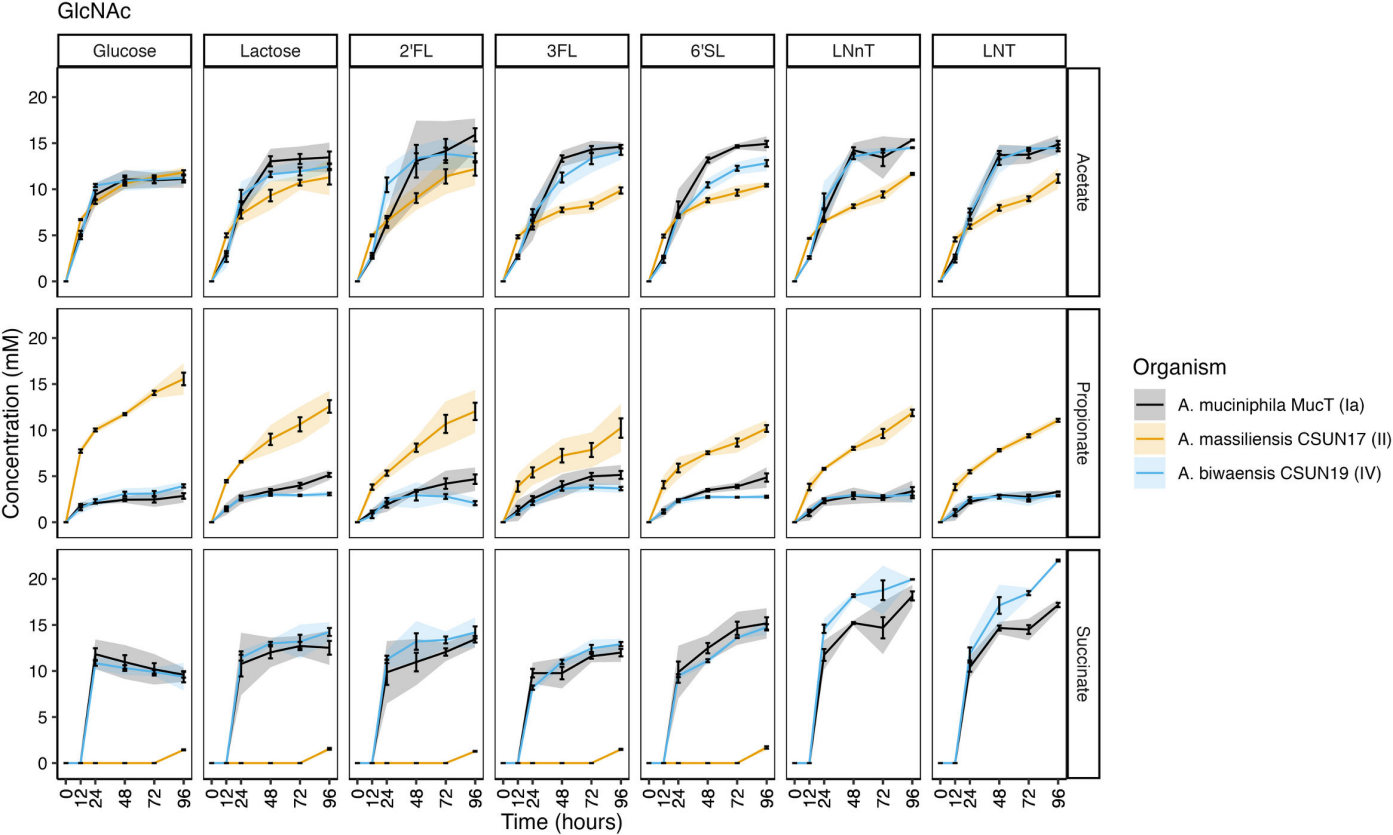
Short-chain fatty acids are produced continuously throughout growth on GlcNAc and HMOs. Concentration of acetate, succinate, and propionate was determined at six time points by HPLC. Error bars represent standard deviation, and shading represents 95% confidence intervals of three technical replicates.

The same SCFAs were detected in cultures grown with a GlcNAc background. Similar to growth on mucin alone, all three organisms had the same amount of acetate accumulated, reaching around 10 mM in the glucose+GlcNAc condition. However, *A. massiliensis* CSUN-17 produced higher levels of propionate and trace levels of succinate under these conditions (Fig. 6). A similar pattern emerged during growth on lactose and all HMOs in a GlcNAc background, in which *A. massiliensis* CSUN-17 had significantly higher levels of propionate, but lower levels of succinate as compared to *A. muciniphila* Muc^T^ and *A. biwaensis* CSUN-19 (*P* < 0.05 at 96 h). Comparing the two high-acetate producers, *A. biwaensis* CSUN-19 and *A. muciniphila* Muc^T^, revealed differences during growth on both LNT and LNnT, where despite equal production of acetate and propionate, *A. biwaensis* CSUN-19 had higher levels of succinate produced.

The increase and then subsequent decrease of succinate that paralleled increasing propionate concentrations in *A. muciniphila* Muc^T^ and *A. biwaensis* CSUN-19 in the mucin but not the GlcNAc media suggested potential vitamin B12 scavenging from mucin (22, 23). To determine if vitamin B12 was present in mucin, we used a *L. leichmannii* ATCC 7830 bioassay to verify the presence of this vitamin. Mucin supported the growth of the vitamin B12 auxotroph, thus confirming the presence of vitamin B12 in mucin preparations (Fig. S5).

## DISCUSSION

Human milk oligosaccharides have intricate interactions with early life colonizers of the human gut microbiota, such as members of the genera *Bifidobacterium*, *Akkermansia*, and *Bacteroides*. Our prior work demonstrated that individual *Akkermansia* species have unique responses to growth across multiple HMOs, partially mediated by differences in the suite of GH enzymes encoded in their genomes (15, 16). In turn, these differences likely affect the ecology of *Akkermansia* in a species-dependent manner. Here, we extend our initial screening of *Akkermansia* growth on HMOs by analyzing the temporal growth dynamics, HMO degradation profiles, and metabolic output across two media backgrounds. We found that *A. biwaensis* CSUN-19 has robust growth in a mucin-media background that is paired with quick degradation of the corresponding HMOs and high production of health-promoting SCFAs. Comparatively, in this same background, the growth of *A. mucinphila* Muc^T^ and *A. massiliensis* CSUN-17 was dependent on the provided HMO, with overall moderate HMO degradation and SCFA production. Growth dynamics changed drastically in a GlcNAc background, wherein *A. muciniphila* Muc^T^ had growth, HMO degradation, and SCFA production patterns similar to *A. biwaensis* CSUN-19. Although *A. massiliensis* CSUN-17 had early growth in this background, the minimal degradation of the HMOs and lower SCFA production suggest overall weak ability for HMO usage under these conditions. Overall, these findings demonstrate that the response to HMOs is species-, HMO-, and media-specific, which may impact the colonization success and overall ecology of *Akkermansia* in the developing infant gut.

Previously, we established HMO degradative capabilities of diverse human-associated *Akkermansia* using 48-h endpoint experiments in a mucin medium background paired with genomic analysis of carbohydrate-active enzymes (15). Here, we extend those findings to include time-resolved growth dynamics and quantitative metabolite production of the same HMOs in three *Akkermansia* species in two media backgrounds. While there are subtle differences in the results between our two experiments (most notably, *A. biwaensis* CSUN-19 consumed more HMOs after 48 h in this experiment than in our previous study), we continue to observe that *A. biwaensis* CSUN-19 has the greatest growth rates and yields when grown on HMOs. While *A. biwaensis* does not appear to be the dominant species of *Akkermansia* in adult human populations (24), it has been isolated from adults across the globe (15, 25, 26). Additionally, the original discovery of this *Akkermansia* lineage (formally known as phylogroup AmIV) was made from metagenomic sequences originating from a population of children aged 2–9 years living in southern California, USA (22, 27). Given the rapid growth and deconstruction of several core HMO structures, future studies should more directly address the prevalence and distribution of *A. biwaensis* in diverse infant populations. Furthermore, given the abundance and diversity of HMO structures across the human population, throughout the lactation period, and dependent on gestation period (20), future studies examining the growth dynamics of more complex HMOs and on pooled HMOs would validate these findings.

*Akkermansia* are mucin-degrading specialists in part because of their inability to aminate fructose-6-phosphate (Fru6P) to form glucosamine-6-phosphate (GlcN6P), a precursor to N-acetyl-D-glucosamine (GlcNAc) (28). Thus, GlcNAc is needed for peptidoglycan biosynthesis (29). Fortunately, mucin glycoproteins are rich in GlcNAc and provide a reliable source for this essential nutrient *in vivo* (30). In the laboratory, this auxotrophy can be overcome by providing mucin or a sugar amine (i.e., GlcNAc or GalNAc) directly in media formulations. Here, in line with previously reported differences across *Akkermansia* species during growth on synthetic versus mucin media (25), we also observe species-specific effects of GlcNAc on growth. For example, *A. massiliensis* CSUN-17 had very similar first-log phase responses to growth in all conditions tested and the largest drop in GlcNAc concentrations within the first 12 h, suggesting the initial growth in the GlcNAc background is using this sugar amine alone rather than the supplemented sugars or HMOs. Ability to use GlcNAc may be dependent on transport mechanisms for this sugar amine: genes that code for GlcNAc transport could not be identified for *A. muciniphila*, suggesting reliance on other sugar transport mechanisms (31). As GlcNAc-supplemented media are suitable for industrial-scale production of *A. muciniphila* (31, 32), understanding how sugar amines differentially support growth may be important for commercial development of other *Akkermansia* species.

HMO degradation by bacteria is often initiated by the removal of the terminal sugar on the non-reducing end of the polysaccharide by exo-acting GH enzymes (33). In the case of the trisaccharide HMOs tested here, this initial hydrolysis would result in free fucose (2′-FL and 3-FL) or sialic acid (6′-SL) and lactose. Indeed, based on detectable levels of fucose, sialic acid, and lactose in spent culture media through time, coupled with the previous characterization of several fucosidase and sialidase enzymes in *Akkermansia* with these activities (33, 34), it appears that *Akkermansia* utilizes this sequential degradation strategy for these HMOs. This is supported by the identification of a gene cluster retaining a putative fucosidase in *A. biwaensis* but not *A. muciniphila* paired with the accumulation of fucose and lactose in culture media (16). However, while degradation of tetrasaccharide HMOs like LNT and LNnT can also be sequential, non-sequential degradation enabled by endo-acting GH enzymes has also been observed in other early life colonizers (reviewed in reference 35). While mediated by different GH enzymes, sequential degradation of LNT and LNnT would result in the trisaccharide lacto-N-triose II (LNT-II) and Gal (35). Sequential degradation of LNT and LNnT is common among *Bifidobacterium* species (35). In contrast, non-sequential degradation could result in the production of two disaccharides; for LNT, these are lacto-N-biose (LNB) and lactose, while for LNnT they would be N-acetyllactosamine (LacNAc) and lactose (36). For example, some species of *Bifidobacterium* use an extracellular endo-acting lacto-N-biosidase (GH20) enzyme to cleave LNT into LNB and lactose (37). When attempting to quantify lactose in our cultures grown on LNT and LNnT, we noticed a small shift in the retention time (<30 s) near where lactose elutes, suggesting the presence of a different sugar, possibly LNT-II, LNB (for LNT), or LacNAc (for LNnT). As we are unable to resolve the identity of this peak, we are unsure of the strategy employed by these *Akkermansia*. It is also worth noting that two recombinant, purified GH16 enzymes from *A. muciniphila* Muc^T^ were shown to remove the terminal glucose from both LNT and LNnT, resulting in the liberation of different trisaccharides (38). While we currently do not know the strategy *Akkermansia* uses to deconstruct these core HMO structures, future studies will determine these strategies.

Across all three organisms grown on 6′-SL, free sialic acid was detected in the culture media with concentrations peaking at 48–72 h, with one exception, *A. massiliensis* CSUN-17 grown in a GlcNAc background. The accumulation of sialic acid has previously been noted during growth on natural pools of HMOs and individual HMOs, as well as on heavily sialylated mucins (15, 17, 33). However, we also observe a decrease in sialic acid in the culture media at later time points for most cultures, suggesting breakdown or use of this compound. In host-associated microorganisms, sialic acids can be degraded for energy conservation (39) or used to decorate the cell surface to evade host immune detection (40). Despite the absence of a canonical N-acylneuraminate (*nan*) catabolic system, it is possible that *Akkermansia* uses a different mechanism to degrade or use sialic acid. Of note, *Akkermansia* possesses an N-acetylneuraminate lyase gene (e.g., Amuc_1946) that may convert sialic acid to N-acetylmannosamine (ManNAc) and pyruvate. From this, the ManNAc can be converted to UDP-N-acetylglucosamine by a UDP-N-acetyl-D-glucosamine 2-epimerase encoded by Amuc_1947. The resulting UDP-N-acetyl-D-glucosamine can be shunted toward cell wall biosynthesis (NAM or NAG) (41). The pyruvate resulting from the initial conversion could supply minimal energy for growth. However, the buildup of sialic acid in the culture medium and consumption of lactose suggest that much of the energy used for growth on 6′-SL likely derives from lactose. Within the context of the gut microbiome, it is possible that the residual sialic acid can be used to support the growth of other gut bacteria (33). It is also possible that instead of converting sialic acid to usable energy, *Akkermansia* incorporates sialic acid into its cell envelope (e.g., lipopolysaccharide and capsular polysaccharide), helping to avoid host immune detection, in line with other host-associated microorganisms (40, 42). Future work is needed to determine the fate of sialic acid liberated from host-produced glycans by *Akkermansia*.

Unexpectedly, all cultures produced large amounts of propionate and no succinate in media containing mucin, whereas in the GlcNAc medium, this phenotype was only observed for *A. massiliensis* CSUN-17. The conversion of propionate to succinate occurs through the vitamin B12-dependent methylmalonyl-CoA synthase (22, 43). Our previous work established that *A. massiliensis* CSUN-17, but neither *A. muciniphila* Muc^T^ nor *A. biwaensis* CSUN-19 synthesizes vitamin B12 *de novo* (22). This is due to the presence of a corrin ring biosynthetic gene cluster present in *A. massiliensis* and *Akkermansia* phylogroup AmIII, but absent in *A. muciniphila* and *A. biwaensis* (22). Notably, although all *Akkermansia* have a vitamin B12-dependent methylmalonyl CoA synthetase involved in propionate metabolism, only *A. massiliensis* and *Akkermansia* phylogroup III have been shown to produce propionate in the absence of exogenous Vitamin B12 (22). However, others have shown that all *Akkermansia* known to date possess a cobamide remodeling gene, CbiR, that allows them to remove and replace the lower ligand of diverse forms of vitamin B12, resulting in production of a usable form (23). Since we do not supplement our media with vitamins, these observations suggest that mucin is providing a form of vitamin B12 accessible to *Akkermansia*. While direct vitamin B12 concentrations have not previously been demonstrated, others have isolated and characterized intrinsic factor, a vitamin B12-binding protein from porcine gastric mucin (44). Vitamin B12 scavenging from mucin may explain previous inconsistencies and batch-to-batch variation in methionine production, another dependent function, while supporting *A. muciniphila* Muc^T^ growth (23). This conversion potentially influences microbe–host interactions, as succinate is involved in immunomodulation (45) and propionate is linked to appetite suppression, potentially mediated through stimulating GLP-1 secretion (46). The ability of some *Akkermansia* (i.e., *A. massiliensis* CSUN-17) to produce propionate in the absence of vitamin B12 may suggest a role in mitigating the effects of obesity and should be further studied. Furthermore, ratios of SCFA production may depend on the carbon source available to *A. muciniphila* during growth, as demonstrated by gene models paired with metabolic end products (18). These outputs may subsequently affect cross-feeding dynamics with other members of the gut microbiota. For example, the liberation of sugars from mucin by *A. muciniphila* Muc^T^ supports the growth of *Eubacterium hallii,* which produces vitamin B12, allowing *A. muciniphila* Muc^T^ to convert succinate to propionate (43). These types of co-culture experiments have revealed an upregulation of genes involved in mucin degradation in *A. muciniphila* during growth with other microorganisms (47). This suggests a propensity for *Akkermansia* to act as a primary degrader for the community. In addition, the *A. muciniphila* co-cultures reveal increased butyrate production in butyrogenic members of the gut microbiota, including *Anaerostipes caccae*, *Roseburia inulinivorans, Faecalibacterium prausnitzii*, and others (33, 43, 47, 48). However, it is unclear whether these scavengers directly consume mucin sugars or act as secondary fermenters consuming acetate released by *Akkermansia*. Furthermore, it is possible that other secondary fermenters of the gut microbiota engage in metabolic cross-feeding by consuming succinate produced by *A. muciniphila* and *A. biwaensis*, as has been observed for other gut microorganisms (49). Therefore, future work aimed at disentangling the metabolic interactions of *Akkermansia* species and other gut microorganisms is essential to holistically understand their impact on human health.

### Conclusion

In conclusion, the ability of *Akkermansia* to fully deconstruct mucin oligosaccharides (34) and a variety of core HMO structures uniquely distinguishes them from other members of the infant gut microbiota. However, like other early-life colonizers, HMO degradation efficiency and potentially the mechanisms employed are species and strain dependent. How these differences impact the colonization success and *in vivo* activity of each *Akkermansia* in mixed gut communities remains largely unknown. Ongoing and future studies will more directly assess how differences across species may impact host–microbe and microbe–microbe interactions in the context of the infant gut microbiome.

## SUPPLEMENTAL MATERIAL

File S1 spectrum.02071-25-s0001.pdf R scripts used to generate figures and statistics.

File S2 spectrum.02071-25-s0002.pdf R scripts used to generate figures and statistics.

File S3 spectrum.02071-25-s0003.pdf R scripts used to generate figures and statistics.

Supplemental figure legends spectrum.02071-25-s0004.docx Legends for Figures S1 to S5.

Figure S1 spectrum.02071-25-s0005.pdf Differences in growth yield are statistically significant across species, HMOs, and media.

Figure S2 spectrum.02071-25-s0006.pdf Relative growth on HMOs is different across species. Growth on the mucin-only culture was subtracted from growth on an individual HMO at 24 hours (A) and 48 hours (B) for each species.

Figure S3 spectrum.02071-25-s0007.pdf Despite the presence of HMOs, in a mucin background, the doubling time and max slope for the first log phase are the same within a species.

Figure S4 spectrum.02071-25-s0008.pdf Decreases in GlcNAc in all strains when grown in this background that is rate-dependent.

Figure S5 spectrum.02071-25-s0009.pdf Mucin contains vitamin B12, supporting the growth of *Lactobacillus leichmanii*.

Tables S1 and S2 spectrum.02071-25-s0010.xlsx Carbon sources and chemicals used in the experiment.

ASM does not own the copyrights to Supplemental Material that may be linked to, or accessed through, an article. The authors have granted ASM a non-exclusive, world-wide license to publish the Supplemental Material files. Please contact the corresponding author directly for reuse.
