# Supplementary material for: Time-resolved growth of diverse human-associated Akkermansia on human milk oligosaccharides
Source: Microbiol Spectr. 2026 Jan 27;14(3):e02071-25. doi: 10.1128/spectrum.02071-25 (PMC12955465; doi:10.1128/spectrum.02071-25)
Supplement: File S1 — R scripts used to generate figures and statistics. [file spectrum.02071-25-s0001.pdf]

GrowthCurveGraphing

Ashwana

02/20/2023

Script Details:

- This script takes the output from the GrowthCurves.Rmd output files (*Cleaned.CSV) and automates graphing* Input files : growth curve csv files from GrowthCurves.Rmd *Output file: png, pdf, or tif file of the growth curve

Load packages

may have to install them first

to install, go to "Tools" > "Install Packages…" and type in the corresponding package name

```
library(reshape2) #melt
library(dplyr) #average, summarise
```

```
##
## Attaching package: 'dplyr'

## The following objects are masked from 'package:stats':
##
##     filter, lag

## The following objects are masked from 'package:base':
##
##     intersect, setdiff, setequal, union
```

```
library(ggplot2) #plot
library(stringr) #add zeroes
library(tidyr) #separate
```

```
##
## Attaching package: 'tidyr'

## The following object is masked from 'package:reshape2':
##
##     smiths
```

```r
library(tidyverse) #rownames_to_column
```

```
## -- Attaching core tidyverse packages ------------------------ tidyverse 2.0.0 --
## v forcats   1.0.0     v readr     2.1.5
## v lubridate 1.9.4     v tibble    3.3.0
## v purrr     1.0.4
```

```
## -- Conflicts ------------------------------------------ tidyverse_conflicts() --
## x dplyr::filter() masks stats::filter()
## x dplyr::lag()    masks stats::lag()
## i Use the conflicted package (<http://conflicted.r-lib.org/>) to force all conflicts to become error
```

```r
library(here) #set the working directory
```

```
## here() starts at /Users/africker/Desktop/DrAsh/02 Data/18 iHMO
```

Load files

Input files needed:

All * CleanAverage.CSV file outputs from GrowthCurves.Rmd.

LOAD and FORMAT files

```r
library(purrr) #map_df

Script.name <- basename(rstudioapi::getSourceEditorContext()$path) #Get the script name - this is to ad

mucin.files <- list.files(here("Mucin","Output","Data"), pattern = "96hr CleanAverage.CSV", full.names
nacetyl.files <- list.files(here("Nacetyl","Output","Data"), pattern = "96hr CleanAverage.CSV", full.na

dd.m <- lapply(1:length(mucin.files), function(idx) {
    d <-read.csv(mucin.files[idx], header = TRUE, sep=",", check.names = FALSE) #read each file
    d.m <- melt(d, c("Sample"), c(4:length(d))) #melt immediately
}) #iterates over file list and reads them all in as CSV
dd.m <- setNames(dd.m, substr(list.files(here("Mucin","Output","Data"), pattern="96hr CleanAverage.CSV"

dd.n <- lapply(1:length(nacetyl.files), function(idx) {
    d <-read.csv(nacetyl.files[idx], header = TRUE, sep=",", check.names = FALSE) #read each file
    d.n <- melt(d, c("Sample"), c(4:length(d))) #melt immediately
}) #iterates over file list and reads them all in as CSV
dd.n <- setNames(dd.n, substr(list.files(here("Nacetyl","Output","Data"), pattern="96hr CleanAverage.CS


mucin.GCs <- map_df(dd.m, ~as.data.frame(.x), .id="GCDate") #Merge the data together
names(mucin.GCs) <- c("GCDate","Sample","hour","OD600")

nacetyl.GCs <- map_df(dd.n, ~as.data.frame(.x), .id="GCDate") #Merge the data together
names(nacetyl.GCs) <- c("GCDate","Sample","hour","OD600")

all.GCs <- rbind(mucin.GCs, nacetyl.GCs)
```

CLEAN data

Remove bad runs and re-graph

```
#Identify and remove the wells do you NOT want to include in the plotting
GCdecontam <- all.GCs[!grepl("23-03-09|24-06-04|23-04-14|23-02-16", all.GCs$GCDate), ] #subset samples

#HMOMucin: No deletions (all replicates look OK)
#HMONacetyl: "23-03-09|24-06-04|23-04-14|23-02-16" (all of these are abnormal)

#Calculate the averages and standard deviation of the cleaned samples
statsClean <- GCdecontam %>%
              group_by(Sample, hour) %>%
              summarise(N=length(OD600),
                        Average=mean(OD600, na.rm = TRUE),
                        SD=sd(OD600, na.rm = TRUE))
```

```
## `summarise()` has grouped output by 'Sample'. You can override using the
## `.groups` argument.
```

```
SelectClean <- statsClean[!grepl("Akk|water|blank", statsClean$Sample, ignore.case = TRUE), ]

SelectClean <- separate(SelectClean, Sample, into = c("Base" ,"Add" , "Org"), sep="\\+", remove = FALSE
SelectClean <- SelectClean %>% mutate_if(is.character, str_trim) #removes whitespace
```

```
## `mutate_if()` ignored the following grouping variables:
## * Column `Sample`
```

```
SelectClean$Add[SelectClean$Add == ""] <- "control"

#Make it pretty
SelectClean$Add <- gsub("Glu", "control", gsub("Lac", "Lactose",gsub("2Fl","2'-FL", gsub("3Fl", "3'-FL"
SelectClean$Base <- gsub("BTTM ", "", gsub("mucin", "Mucin", gsub("Nacetyl", "GlcNAc",SelectClean$Base)
SelectClean$Base <- factor(SelectClean$Base, levels=c("Mucin", "GlcNAc"))
SelectClean$Add <- factor(SelectClean$Add, levels=c("control", "Lactose", "2'-FL", "3'-FL", "6'-SL", "L
SelectClean$Org <- gsub("Type", "A. muciniphila MucT (Ia)",gsub("CSUN17", "A. massiliensis CSUN17 (II)"
SelectClean$Org <- factor(SelectClean$Org, levels=c("A. muciniphila MucT (Ia)", "A. massiliensis CSUN17

#Create color palettes
cbbPalette <- c("#000000", "#E69F00", "#56B4E9", "#009E73", "#F0E442", "#0072B2", "#D55E00", "#CC79A7")

#Plot the cleaned and selected samples
ggplot(SelectClean, aes(as.numeric(hour), Average, group=Sample)) + #can choose data to plot (all=stats
  geom_line(aes(group=Sample, color=Org), size=1.5) + #Make it a line graph
  geom_ribbon(aes(ymin=Average-SD, ymax=Average+SD, fill=Org), color=NA, alpha=0.3) + #ribbon instead o
  labs(x="Time (Hours)", y="Absorbance at 600 nm", title = NULL, caption=NULL) + #Set axis and title la
  facet_grid(Add~Base) +
  scale_fill_manual(values = cbbPalette, name = "Organism") +
  scale_color_manual(values=cbbPalette, name="Organism") +
  theme_bw() + #Change background to a black and white
  theme(panel.grid = element_blank(), #Remove the x- and y- lines on the graph
```

```
        axis.text.x = element_text(angle=90, size=14, color="black"),
        axis.title.x = element_text(size=16, color="black"),
        axis.text.y = element_text(size=14, color="black"),
        axis.title.y = element_text(size=16, color="black"),
        legend.position = "right",
        legend.text = element_text(size=14, color="black"),
        legend.title = element_text(size=14, color="black"),
        strip.background =element_rect(fill="white"),
        panel.spacing.x = unit(2, "lines"),
        strip.text.x = ggtext::element_textbox_simple(width = unit(1, "npc"),
                                                height = unit(2, "lines"),
                                                colour = 'black',
                                                size = 16,
                                                hjust = 0.5,
                                                vjust = 0.5,
                                                halign = 0.5,
                                                valign = 0.5),
        strip.text.y = ggtext::element_textbox_simple(width = unit(1, "npc"),
                                                height = unit(2, "lines"),
                                                colour = 'black',
                                                size = 14,
                                                hjust = 0.5,
                                                vjust = 0.5,
                                                halign = 0.5,
                                                valign = 0.5,
                                                orientation = "right-rotated")) +
  scale_x_continuous(expand = c(0, 0), limits = c(NA,96), breaks = seq(0, 100, by = 10)) +
  scale_y_continuous(breaks = seq(0, 2.0, by = 1.0))  #Start the graph at the y axis (otherwise it star
```

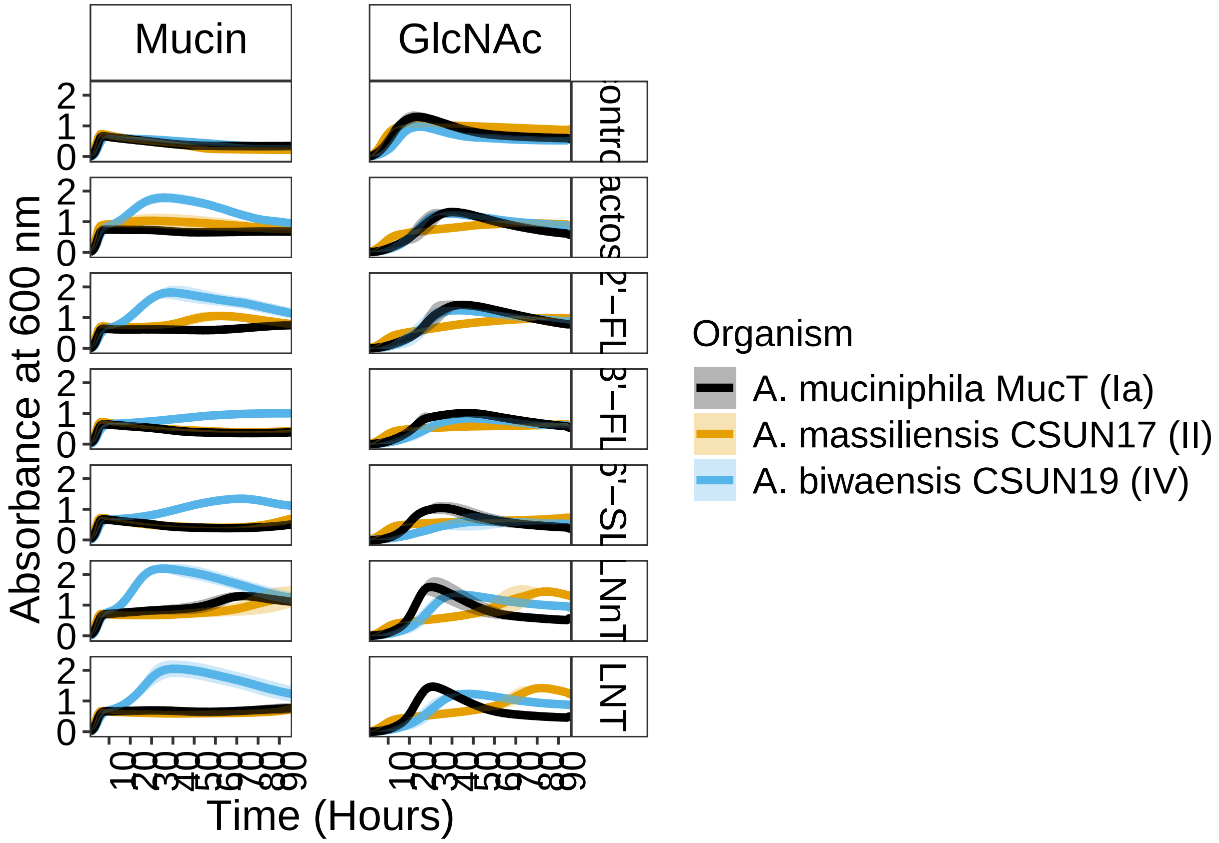


```
#ggsave(here("CombinedOut",paste("HMO.96h.CleanCurves",format(Sys.time(), "%Y%m%d%H%M"),"png", sep="."))
```

CALCULATE: (run full series)

MAX OD and specific time points

```
library(ggh4x) #separate

#Identify and remove the wells do you NOT want to include in the plotting
GCdecontam <- all.GCs[!grepl("23-03-09|24-06-04|23-04-14|23-02-16", all.GCs$GCDate), ] #subset samples

#HMOMucin: No deletions (all replicates look OK)
#HMONacetyl: "23-03-09|24-06-04|23-04-14|23-02-16" (all of these are abnormal)

#Calculate Max OD
normGCMax <- GCdecontam %>%
  group_by(Sample, GCDate) %>%
  summarise(MaxOD = max(OD600), #calculate max OD
            max.hr = as.numeric(hour[which.max(OD600)])) #Time of max OD
```

```
## `summarise()` has grouped output by 'Sample'. You can override using the
## `.groups` argument.
```

```r
#Change to GCdecontam if samples were removed!

#Pull out data from specific timepoints
normGCtimepts <- GCdecontam[as.numeric(GCdecontam$hour) %in% c('24','48', '72','94'),]
normGCtimepts <- reshape(normGCtimepts, idvar = c("Sample", "GCDate"), timevar = "hour", direction = "wi

#Combine data frames
GCMaxTimePts <- merge(x=normGCMax, y=normGCtimepts, by=c("Sample","GCDate"))[]


GCMaxDelta <- separate(GCMaxTimePts, Sample, into = c("Base" ,"HMO", "Org"), sep="\\+", remove = FALSE)
GCMaxDelta <- GCMaxDelta %>% mutate_if(is.character, str_trim) #removes whitespace
colnames(GCMaxDelta) <- gsub(" ","",colnames(GCMaxDelta)) #removes whitespace in column headers
GCMaxDelta <- GCMaxDelta[!grepl("Akk|water|blank", GCMaxDelta$Sample, ignore.case = TRUE),]

#Calculate change in OD (delta)
GCMaxDelta$HMO[GCMaxDelta$HMO == ""] <- "Control"
GCMaxDelta$HMO <- gsub("Glu", "Control",GCMaxDelta$HMO)

GCMaxDelta <- GCMaxDelta %>%
  group_by(Org, GCDate) %>%
  mutate(delta24 = OD600.24-OD600.24[HMO == "Control"]) %>%
  mutate(delta48 = OD600.48-OD600.48[HMO == "Control"])


GCMaxDelta <- GCMaxDelta[ -c(2:4) ] #remove separated columns
```

LAG phase using "growthrates"

```r
# Need this additional package for calculating lag.
## This package can also be used to calculate growth rates, but I don't like it, so I wrote my own (bel
library(growthrates)
```

```
## Loading required package: lattice

## Loading required package: deSolve
```

```r
#Identify and remove the wells do you NOT want to include in the plotting

#Identify and remove the wells do you NOT want to include in the plotting
GCdecontam <- all.GCs[!grepl("23-03-09|24-06-04|23-04-14|23-02-16", all.GCs$GCDate), ] #subset samples 

#HMOMucin: No deletions (all replicates look OK)
#HMONacetyl: "23-03-09|24-06-04|23-04-14|23-02-16" (all of these are abnormal)

GCdecontam <- GCdecontam[!grepl("Akk|water|blank", GCdecontam$Sample, ignore.case = TRUE), ] #remove wa
GCdecontam <- GCdecontam[GCdecontam$OD600 > 0, ]
GCdecontam <- transform(GCdecontam, hour = as.numeric(hour))


linearGC <- all_easylinear(OD600 ~ hour  | Sample + GCDate , data = GCdecontam, h = 7)
```

```
linearGCres <- results(linearGC) #make a data frame to place the results: y0(where the line crosses axi
linearGCres <- linearGCres %>%
  rownames_to_column(var = "Rownames") %>%
  separate(Rownames,sep = "\\:",into = c("SampleID2","GCDate"))

linearGCres <- linearGCres[ -c(1, 4:6, 8) ] #remove separated columns
### growthcurves REFERENCE: https://cran.r-project.org/web/packages/growthrates/vignettes/Introduction.
```

GROWTH RATE using rolling slope

```
# Need this additional package for calculating the rolling slope (rollapplyr)
library(zoo)
```

```
##
## Attaching package: 'zoo'

## The following objects are masked from 'package:base':
##
##     as.Date, as.Date.numeric
```

```
slope <- . %>% { cov(.[, 2], .[, 1]) / var(.[, 2])}

GC.slope <- GCdecontam %>%
       group_by(Sample, GCDate) %>%
       mutate(slope = rollapplyr(cbind(OD600, hour), 3, slope, by.column = FALSE, fill = NA),
              dt = (log(2))/slope) %>%
       ungroup

GC.slope.max <- GC.slope %>%
       group_by(Sample, GCDate) %>%
       summarise(mu = max(slope, na.rm=TRUE),
                 dt = (log(2))/max(slope, na.rm=TRUE))
```

```
## `summarise()` has grouped output by 'Sample'. You can override using the
## `.groups` argument.
```

```
##CHECK WITH DR FLORES ##
## This still needs to be double checked with Dr. Flores' hand-calculations, but I think it's right :)
```

MERGE data frames

```
library(rstatix) #tukey's
```

```
##
## Attaching package: 'rstatix'
```

```
## The following object is masked from 'package:stats':
##
##     filter
```

```
library(ggpubr) #stat_pval_manual
library(ggh4x) #facet_nested

data.df <- inner_join(GCMaxDelta, linearGCres, by = c("Sample", "GCDate")) %>%
  left_join(GC.slope.max, by = c("Sample", "GCDate")) %>%
  separate(Sample, into = c("Base" ,"HMO", "Org"), sep="\\+", remove = FALSE) %>%
  mutate_if(is.character, str_trim) #removes whitespace
```

```
## `mutate_if()` ignored the following grouping variables:
## * Column `GCDate`
```

```
data.df$HMO[data.df$HMO == ""] <- "Control"
data.df$HMO <- gsub("Glu", "Control",
                    gsub("Lac", "Lactose",
                         gsub("2Fl","2'-FL",
                              gsub("3Fl", "3'-FL",
                                   gsub("6Sl", "6'-SL", data.df$HMO)))))
data.df <- data.df %>%
  group_by(Base) %>%
  mutate(HMO = factor(HMO, levels = c("Control", "Lactose", "2'-FL", "3'-FL", "6'-SL", "LNnT", "LNT")))


data.df$Org <- gsub("Type", "MucT (Ia)",
                    gsub("CSUN17", "CSUN17 (II)",
                         gsub("CSUN19", "CSUN19 (IV)",data.df$Org)))
data.df$Org <- factor(data.df$Org, levels=c("MucT (Ia)", "CSUN17 (II)", "CSUN19 (IV)"))


# data.df$Org <- gsub("Type", "MucT",
#                     gsub("CSUN17", "CSUN17",
#                          gsub("CSUN19", "CSUN19",data.df$Org)))
# data.df$Org <- factor(data.df$Org, levels=c("MucT", "CSUN17", "CSUN19"))


data.df$Base <- gsub("BTTM Mucin", "Mucin",
                    gsub("BTTM Nacetyl", "GlcNAc", data.df$Base))
data.df$Base <- factor(data.df$Base, levels=c("Mucin", "GlcNAc"))

#data.df <- data.df[grepl("Mucin", data.df$Base, ignore.case = TRUE), ]

data.df.stat <- data.df %>%
              group_by(Sample, Base, HMO, Org) %>%
              summarise(Lag = mean(lag),
                        LagSD = sd(lag),
                        Max = mean(MaxOD),
                        MaxSD = sd(MaxOD),
                        MaxHr = mean(max.hr),
                        MaxHrSD = sd(max.hr),
                        Slope = mean(mu),
```

```
                        SlopeSD = sd(mu),
                        h48 = mean(OD600.48),
                        h48SD = sd(OD600.48),
                        delt24 = mean(delta24),
                        delt24SD = sd(delta24),
                        delt48 = mean(delta48),
                        delt48SD = sd(delta48)) %>%
                rename(., MaxOD = Max) %>%
                rename(., MaxODSD = MaxSD) %>%
                rename(., OD600.48 = h48) %>%
                rename(., OD600.48SD = h48SD) %>%
                rename(., delta24 = delt24) %>%
                rename(., delta24SD = delt24SD) %>%
                rename(., delta48 = delt48) %>%
                rename(., delta48SD = delt48SD) %>%
                ungroup()
```

```
## `summarise()` has grouped output by 'Sample', 'Base', 'HMO'. You can override
## using the `.groups` argument.
```

CHOOSE Variables to graph

```
var <- "MaxOD" #set this to be the same as what you use in stat.test (can be OD600.48, MaxOD, max.hr, o
varSD <- paste(var,"SD",sep="")
```

STATISTICAL tests

```
# #Test if the data is normally distributed (shaprio test) p>0.05 = normal distribution (significance =
data.df %>%
  ungroup() %>%
   shapiro_test(lag)
```

```
## # A tibble: 1 x 3
##   variable statistic        p
##   <chr>        <dbl>    <dbl>
## 1 lag          0.758 2.84e-14
```

```
#If significant, run Kruskal-Wallis
##BY HMO
tmp <- data.df %>%
  dplyr::group_by(Base, Org) %>%
  kruskal_test(lag ~ HMO)
##BY Org
tmp <- data.df %>%
  dplyr::group_by(HMO, Base) %>%
  kruskal_test(delta48 ~ Org)
##BY Media
tmp <- data.df %>%
```

```
  dplyr::group_by(HMO, Org) %>%
  kruskal_test(delta48 ~ Base)
```

GRAPH

GRAPH Max

```
stat.test <- data.df %>%
  dplyr::group_by(HMO, Base) %>%
  dunn_test(MaxOD ~ Org, p.adjust.method = "bonferroni") %>% ##NOTE: Change the values here that you're
  dplyr::filter(!is.na(p.adj)) %>%
  ungroup() %>%
  add_significance("p.adj") %>%
  add_x_position(x = "Org") %>% #This must be the same as the stat variable and same as X in ggplot
  add_y_position(fun = "max", scales = "fixed") #Add x and y position to graph significance

data.df.stat %>%
  ggplot( aes(x = Org, y = get(var))) + #x should be the same as x_position in stat.test
    facet_nested(.~HMO+Base, space="free", scales="free_x") + #create sub-plots
    geom_col(fill="black")+
    geom_errorbar(aes(ymin = get(var) - ifelse(get(var) < 0, get(varSD), 0),
        ymax = get(var) + ifelse(get(var) > 0, get(varSD), 0))) +  #unidirectional errorbar
    stat_pvalue_manual(stat.test, label="p.adj.signif", hide.ns = TRUE, tip.length = 0 ) + #add statist
    theme_classic() + #get rid of the grey background
    theme(axis.text.x = element_text(angle = 90, vjust = 0.5, hjust=1, size = 12),
          strip.background =element_rect(fill="white"))+
    scale_fill_manual(values=c("white", "#C0C0C0")) +
   labs(x= NULL, y = paste("OD", "(600nm)"), title = var, caption=Script.name)
```

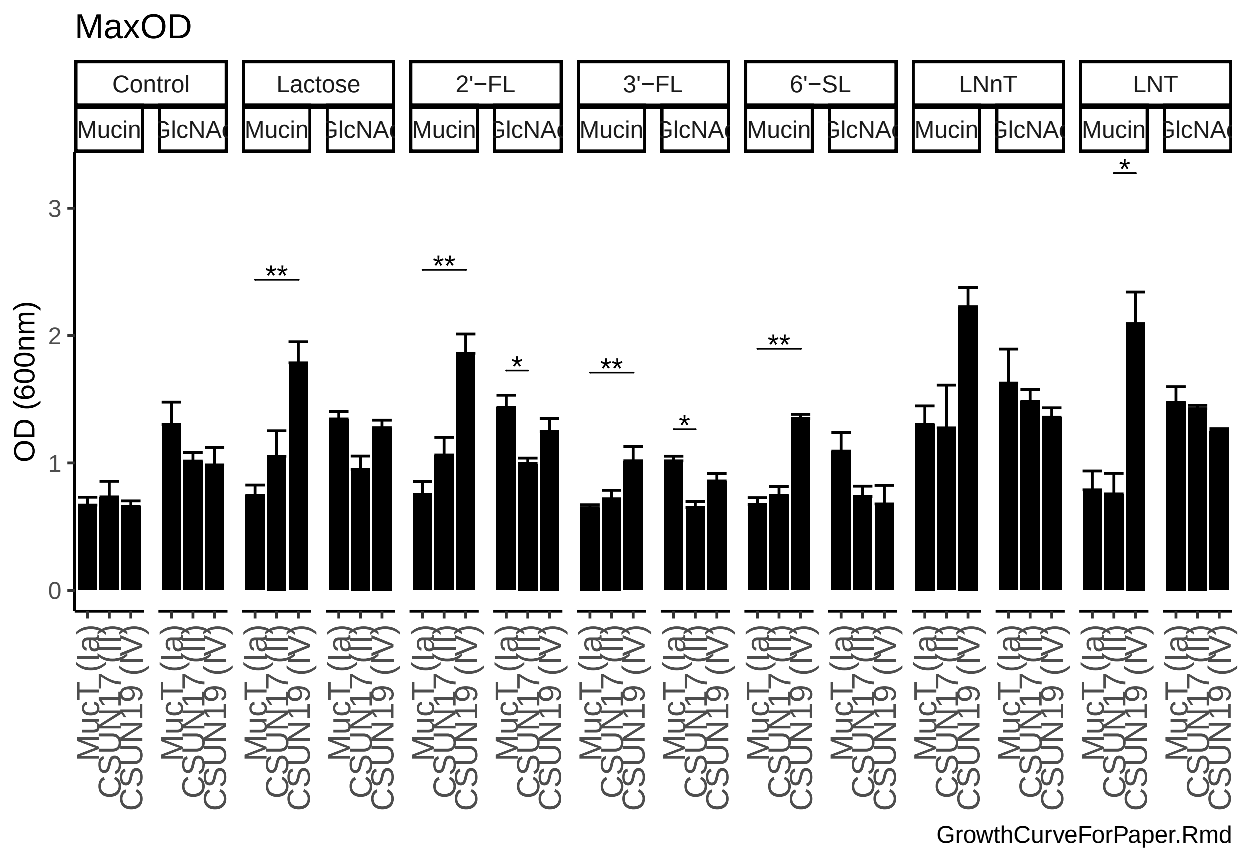

GRAPH Deltas

GRAPH Lag

Save the graph

```
#ggsave(here("CombinedOut",paste("HMO","dunn",var,format(Sys.time(), "%Y%m%d%H%M"),"png", sep=".")), wi
```
