# Supplementary material for: Time-resolved growth of diverse human-associated Akkermansia on human milk oligosaccharides
Source: Microbiol Spectr. 2026 Jan 27;14(3):e02071-25. doi: 10.1128/spectrum.02071-25 (PMC12955465; doi:10.1128/spectrum.02071-25)
Supplement: File S2 — R scripts used to generate figures and statistics. [file spectrum.02071-25-s0002.pdf]

HPLC_Parsing

Ashwana

2023-09-01

LOAD libraries

```
lapply(names(sessionInfo()$loadedOnly), require, character.only = TRUE)
```

```
## Loading required package: compiler
```

```
## Loading required package: fastmap
```

```
## Loading required package: cli
```

```
## Loading required package: tools
```

```
## Loading required package: htmltools
```

```
## Loading required package: rstudioapi
```

```
## Loading required package: yaml
```

```
## Loading required package: rmarkdown
```

```
## Loading required package: knitr
```

```
## Loading required package: xfun
```

```
##
## Attaching package: 'xfun'
```

```
## The following objects are masked from 'package:tools':
##
##     file_ext, Rcmd
```

```
## The following object is masked from 'package:cli':
##
##     tree
```

```
## The following object is masked from 'package:base':
##
##     attr

## Loading required package: digest

## Loading required package: rlang

## Loading required package: evaluate

## [[1]]
## [1] TRUE
##
## [[2]]
## [1] TRUE
##
## [[3]]
## [1] TRUE
##
## [[4]]
## [1] TRUE
##
## [[5]]
## [1] TRUE
##
## [[6]]
## [1] TRUE
##
## [[7]]
## [1] TRUE
##
## [[8]]
## [1] TRUE
##
## [[9]]
## [1] TRUE
##
## [[10]]
## [1] TRUE
##
## [[11]]
## [1] TRUE
##
## [[12]]
## [1] TRUE
##
## [[13]]
## [1] TRUE
```

```r
invisible(lapply(paste0('package:', names(sessionInfo()$otherPkgs)), detach, character.only=TRUE, unloa
```

```
## Warning: 'evaluate' namespace cannot be unloaded:
##   namespace 'evaluate' is imported by 'rmarkdown' so cannot be unloaded
```

```
## Warning: 'rlang' namespace cannot be unloaded:
##   namespace 'rlang' is imported by 'htmltools' so cannot be unloaded

## Warning: 'digest' namespace cannot be unloaded:
##   namespace 'digest' is imported by 'htmltools' so cannot be unloaded

## Warning: 'xfun' namespace cannot be unloaded:
##   namespace 'xfun' is imported by 'knitr' so cannot be unloaded

## Warning: 'knitr' namespace cannot be unloaded:
##   namespace 'knitr' is imported by 'rmarkdown' so cannot be unloaded
```

```r
library(tidyr) #separate
library(ggplot2) #plot
library(rstatix) #running statistics
```

```
##
## Attaching package: 'rstatix'

## The following object is masked from 'package:stats':
##
##     filter
```

```r
library(ggpubr) #plotting statistics
library(dplyr) #select and combine data
```

```
##
## Attaching package: 'dplyr'

## The following objects are masked from 'package:stats':
##
##     filter, lag

## The following objects are masked from 'package:base':
##
##     intersect, setdiff, setequal, union
```

```r
library(purrr) # map_df
library(ggh4x) #facet with different y axis labels
library(stringr) #partial string match
library(here) #set working directory
```

```
## here() starts at /Users/africker/Desktop/DrAsh/02 Data/18 iHMO/Nacetyl
```

READ in data

```
script.name <- basename(rstudioapi::getSourceEditorContext()$path) #Assign the script name

SCFA.files <- list.files(here("Data"), pattern = "Timecourse.+v4.csv", full.names = TRUE) #Finds all av

dd.m <- lapply(1:length(SCFA.files), function(idx) {
    # load the file
    d <-read.csv(SCFA.files[idx], header = TRUE, sep=",", check.names = FALSE)
}) #iterates over file list and reads them all in as CSV
dd.m <- setNames(dd.m, substr(list.files(here( "Data"), pattern="Timecourse.+v4.csv"), 24,32)) #Gives e

SCFA <- map_df(dd.m, ~as.data.frame(.x), .id="Date") #Merge the data together
#write.csv(unique(SCFA$Sample), file=here("Data","HMO_Sample_Metadata.csv"), row.names = FALSE) #Write

meta <- read.csv(here("Data","HMO_Sample_GlcNAc_Metadata.csv"), header=T)
meta$Organism <- gsub("-","", meta$Org)

#Combine list into data and add metadata
SCFA.df.meta<-merge(SCFA,meta,by="Sample")
SCFA.df.meta$Concent <- as.numeric(SCFA.df.meta$Concent)
SCFA.df.meta$Time <- as.character(SCFA.df.meta$Time)

#Remove specific re-run samples
SCFA.df.meta <- SCFA.df.meta %>% filter(!(str_detect(`Injection Acquired Date`, '2024-07-07') & Sample=
SCFA.df.meta <- SCFA.df.meta %>% filter(!(str_detect(`Injection Acquired Date`, '2024-07-08') & Sample=

#Select name of negative controls
Neg <- c("Neg Ctrl")
```

CONVERT Data:

Select SCFA, HMOs, etc.

```
Cpds.df <- SCFA.df.meta[grep(".", SCFA.df.meta$Name), ]
SCFA.df <- Cpds.df[grep("Acetate|Propionate|Succinate|1,2-propanediol", Cpds.df$Name, ignore.case=TRUE)
HMOs.df <- Cpds.df[grep("2'-FL|3'-FL|6'-SL|LNT|LNnT", Cpds.df$Name, ignore.case=TRUE),]
HMOs.df <- HMOs.df[!grepl("none", HMOs.df$HMO),]
Lactose.df<-Cpds.df %>% filter(HMO == "Lactose" & Name == "Lactose")
HMOs.df.2 <- rbind(HMOs.df, Lactose.df)
Sugars.df <- Cpds.df[grep("Lactose|Fucose|Sialic Acid|N-acetyl-D-glucosamine|Glucose", Cpds.df$Name),]


Cpds.clean <- Cpds.df %>%
  group_by(Name, HMO, Org, Replicate, Organism, Base) %>%
  tidyr::complete(Time = c("0", "12","24", "48", "72", "96")) %>% #Fill empty values with NA
  mutate(ConcSubtr = Concent-Concent[Time == "0"]) #Subtract time zero values
#         across(where(is.numeric), ~replace(., is.na(.), 0))) #Set NA value to zero

SCFA.clean <- SCFA.df %>%
  group_by(Name, HMO, Org, Replicate, Organism, Base) %>%
  tidyr::complete(Time = c("0", "12", "24", "48", "72", "96")) %>% #Fill empty values with NA
```

```
  mutate(across(where(is.numeric), ~replace(., is.na(.), 0))) %>%
  mutate(ConcSubtr = Concent-Concent[Time == "0"]) #Subtract time zero values

HMO.negs<- as.data.frame(HMOs.df.2[(HMOs.df.2$Organism %in% Neg),]) %>%
  group_by(Name, HMO, Org, Replicate, Organism, Base) %>%
  tidyr::complete(Time = c("0", "48", "96")) %>% #Fill empty values with NA
  mutate(ConcSubtr = 100*(Concent/Concent[Time == "0"])) #Get percent used

HMO.clean <- as.data.frame(HMOs.df.2[!(HMOs.df.2$Organism %in% Neg),]) %>%
  group_by(Name, HMO, Org, Replicate, Organism, Base) %>%
  tidyr::complete(Time = c("0", "12", "24", "48", "72", "96")) %>% #Fill empty values with NA
  mutate(across(where(is.numeric), ~replace(., is.na(.), 0))) %>%
  mutate(ConcSubtr = 100*(Concent/Concent[Time == "0"])) #Get percent used

HMO.clean.negs <- rbind(HMO.negs, HMO.clean)

Sugars.clean <- Sugars.df %>%
  group_by(Name, HMO, Org, Replicate, Organism, Base) %>%
  tidyr::complete(Time = c("0", "12", "24", "48", "72", "96")) %>% #Fill empty values with NA
  mutate(across(where(is.numeric), ~replace(., is.na(.), 0))) %>%
  mutate(ConcSubtr = Concent-Concent[Time == "0"]) #Subtract time zero values

Cpds.clean$HMO <- gsub("'-", "", Cpds.clean$HMO)
#Cpds.clean$Name <- gsub(",2-", "2", Cpds.clean$Name)

SCFA.clean$HMO <- gsub("3'FL", "3FL", gsub("-","", SCFA.clean$HMO))
HMO.clean.negs$HMO <- gsub("3'FL", "3FL", gsub("-","", HMO.clean.negs$HMO))
Sugars.clean$HMO <- gsub("3'FL", "3FL", gsub("-","", Sugars.clean$HMO))
```

Calculate Averages

```
#Remove samples that aren't triplicates
Cpds.clean.samples <- as.data.frame(Cpds.clean[!(Cpds.clean$Organism %in% Neg),])  %>%
  group_by(Name, HMO, Time, Org) %>%
  mutate(N=length(ConcSubtr))  %>%
  filter(N>=3) %>%
  ungroup()

SCFA.clean.samples <- as.data.frame(SCFA.clean[!(SCFA.clean$Organism %in% Neg),])  %>%
  group_by(Name, HMO, Time, Org) %>%
  mutate(N=length(ConcSubtr))  %>%
  filter(N>=3) %>%
  ungroup()

HMO.clean.samples <- HMO.clean.negs %>%
  as.data.frame(HMO.clean[!(HMO.clean$Organism %in% Neg),])  %>% #Remove negative controls
  group_by(Name, HMO, Time, Org) %>%
  mutate(N=length(ConcSubtr))  %>%
  filter(N>=3) %>% #This will remove negative controls if left in
  ungroup()
```

```
Sugars.clean.samples <- as.data.frame(Sugars.clean[!(Sugars.clean$Organism %in% Neg),])  %>%
  group_by(Name, HMO, Time, Org) %>%
  mutate(N=length(ConcSubtr))  %>%
  filter(N>=3) %>%
  ungroup()


#Calculate average and standard deviation on data
Cpds.stats <- Cpds.clean.samples %>%
  group_by(Name, HMO, Org, Base, Time) %>%
  summarise(N=length(ConcSubtr),
            Average=mean(ConcSubtr),
            SD=sd(ConcSubtr)) %>%
  arrange(desc(Average))%>%
  ungroup() %>%
  na.omit()
```

```
## `summarise()` has grouped output by 'Name', 'HMO', 'Org', 'Base'. You can
## override using the `.groups` argument.
```

```
SCFA.stats <- SCFA.clean.samples %>%
  group_by(Name, HMO, Org, Base, Time) %>%
  summarise(N=length(ConcSubtr),
            Average=mean(ConcSubtr),
            SD=sd(ConcSubtr)) %>%
  arrange(desc(Average))%>%
  ungroup() %>%
  na.omit() %>%
  mutate(Time=as.numeric(Time))
```

```
## `summarise()` has grouped output by 'Name', 'HMO', 'Org', 'Base'. You can
## override using the `.groups` argument.
```

```
HMO.stats <- HMO.clean.samples %>%
  group_by(Name, HMO, Org, Base, Time) %>%
  summarise(N=length(ConcSubtr),
            Average=mean(ConcSubtr),
            SD=sd(ConcSubtr)) %>%
  arrange(desc(Average)) %>%
  ungroup() %>%
  filter(is.finite(Average)) %>% #Remove samples that are "infinite"
  mutate(Time=as.numeric(Time))
```

```
## `summarise()` has grouped output by 'Name', 'HMO', 'Org', 'Base'. You can
## override using the `.groups` argument.
```

```
Sugars.stats <- Sugars.clean.samples %>%
  group_by(Name, HMO, Org, Base, Time) %>%
  summarise(N=length(ConcSubtr),
            Average=mean(ConcSubtr),
            SD=sd(ConcSubtr)) %>%
```

```r
  arrange(desc(Average))%>%
  ungroup() %>%
  na.omit()
```

```
## `summarise()` has grouped output by 'Name', 'HMO', 'Org', 'Base'. You can
## override using the `.groups` argument.
```

```r
Nacetyl.stats <- Sugars.clean.samples %>%
  group_by(Name, HMO, Org, Base, Time) %>%
  summarise(N=length(Concent),
            Average=mean(Concent),
            SD=sd(Concent)) %>%
  arrange(desc(Average))%>%
  ungroup() %>%
  na.omit()
```

```
## `summarise()` has grouped output by 'Name', 'HMO', 'Org', 'Base'. You can
## override using the `.groups` argument.
```

GRAPH

Set palette

```r
#Set colorblind palette
cbbPalette <- c("#000000", "#E69F00", "#56B4E9", "#009E73", "#F0E442", "#0072B2", "#D55E00", "#CC79A7")
```

##Graph SCFA data

```r
SCFA.stats.2 <- SCFA.stats[!grepl("propanediol",SCFA.stats$Name),] %>%
    mutate(Time=as.numeric(Time))
#Set order of facets so they align with mucin
SCFA.stats.2$Org <- gsub("MucT", "A. muciniphila MucT (Ia)",gsub("CSUN-17", "A. massiliensis CSUN17 (II
SCFA.stats.2$Org <- factor(SCFA.stats.2$Org, levels=c("A. muciniphila MucT (Ia)", "A. massiliensis CSUN
SCFA.stats.2$HMO_f = factor(SCFA.stats.2$HMO, levels=c("Glucose", "Lactose", "2'FL", "3FL", "6'SL", "LN

#Plot graph
SCFA.stats.2 %>%
  ggplot(aes(Time, Average, group=Org)) +
  geom_line(aes(color=Org), na.rm = TRUE) + #bar graph
  geom_errorbar(aes(ymin = Average - SD,
                    ymax = Average + SD), width=4) + #errorbar
#  ggh4x::facet_grid2(HMO ~ Name, scales = "free_y", independent = "y") +
  facet_grid(Name~HMO_f, scales="free_y", space="free_x") +  #create sub-plots
  theme_classic() + #get rid of the grey background
  theme(axis.text=element_text(color="black"),
        axis.text.x = element_text(angle = 90, vjust = 0.5, hjust=1),
        panel.border = element_rect(color="black", fill=NA),
        strip.background =element_rect(fill="white"))+
  scale_color_manual(values = cbbPalette, name = "Organism") + #set plot colors
```

```
  scale_x_continuous(breaks = SCFA.stats.2$Time, labels = SCFA.stats.2$Time) +
  labs(x= "Time (hours)", y = "Concentration (mM)", title=NULL, subtitle = "GlcNAc",
       caption=NULL) + #add axis labels
  #caption=paste("made using", script.name, "\ngenerated on", Sys.time(), sep=" ")) +  # add caption th
  ylim(-0.6,22.1)
```

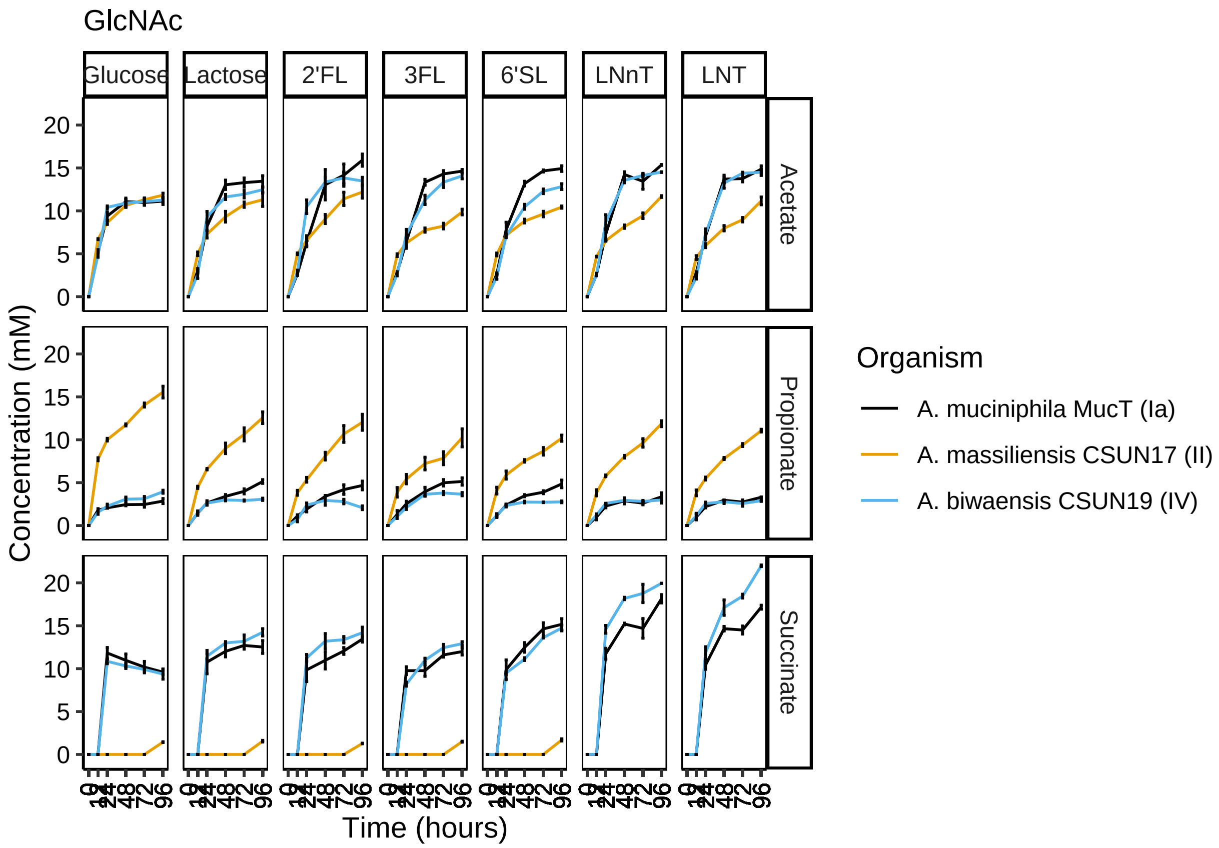


```
#ggsave(here("Output","Figures",paste("iHMO_Timecourse_SCFAs",format(Sys.time(), "%Y-%m-%d-%H-%M"),"png
```

##Graph HMO data

```
HMO.stats.2 <- HMO.stats%>%
    mutate(Time=as.numeric(Time))
HMO.stats.2$Org <- gsub("MucT", "A. muciniphila MucT (Ia)",gsub("CSUN-17", "A. massiliensis CSUN17 (II)
HMO.stats.2$Org <- factor(HMO.stats.2$Org, levels=c("A. muciniphila MucT (Ia)", "A. massiliensis CSUN17
HMO.stats.2$HMO_f <- factor(HMO.stats.2$HMO, levels=c("Glucose", "Lactose", "2'FL", "3FL", "6'SL", "LNn

#Plot graph
HMO.stats.2 %>%
  ggplot( aes(Time, Average, group=Org)) +
  geom_line(aes(color=Org), na.rm = TRUE) + #bar graph
  geom_errorbar(aes(ymin = Average - SD,
                    ymax = Average + SD), width=3) + #unidirectional errorbar
  facet_grid(HMO_f~., scales="free_x", space="free_x") +  #create sub-plots
  theme_classic() + #get rid of the grey background
```

```
theme(axis.text.x = element_text(angle = 90, vjust = 0.5, hjust=1, size = 8),
      panel.border = element_rect(color="black", fill=NA),
      strip.background =element_rect(fill="white"),
      strip.text.x = ggtext::element_textbox_simple(width = unit(1, "npc"),
                                                    height = unit(1, "lines"),
                                                    colour = 'black',
                                                    size = 7,
                                                    hjust = 0.5,
                                                    vjust = 0.5,
                                                    halign = 0.5,
                                                    valign = 0.5)) +
scale_color_manual(values = cbbPalette, name = "Organism") + #set plot colors
scale_x_continuous(breaks = HMO.stats$Time, labels = HMO.stats$Time) +
labs(x= "Time (hours)", y = "Percent remaining", title=NULL, subtitle = "GlcNAc",
     caption=NULL)
```

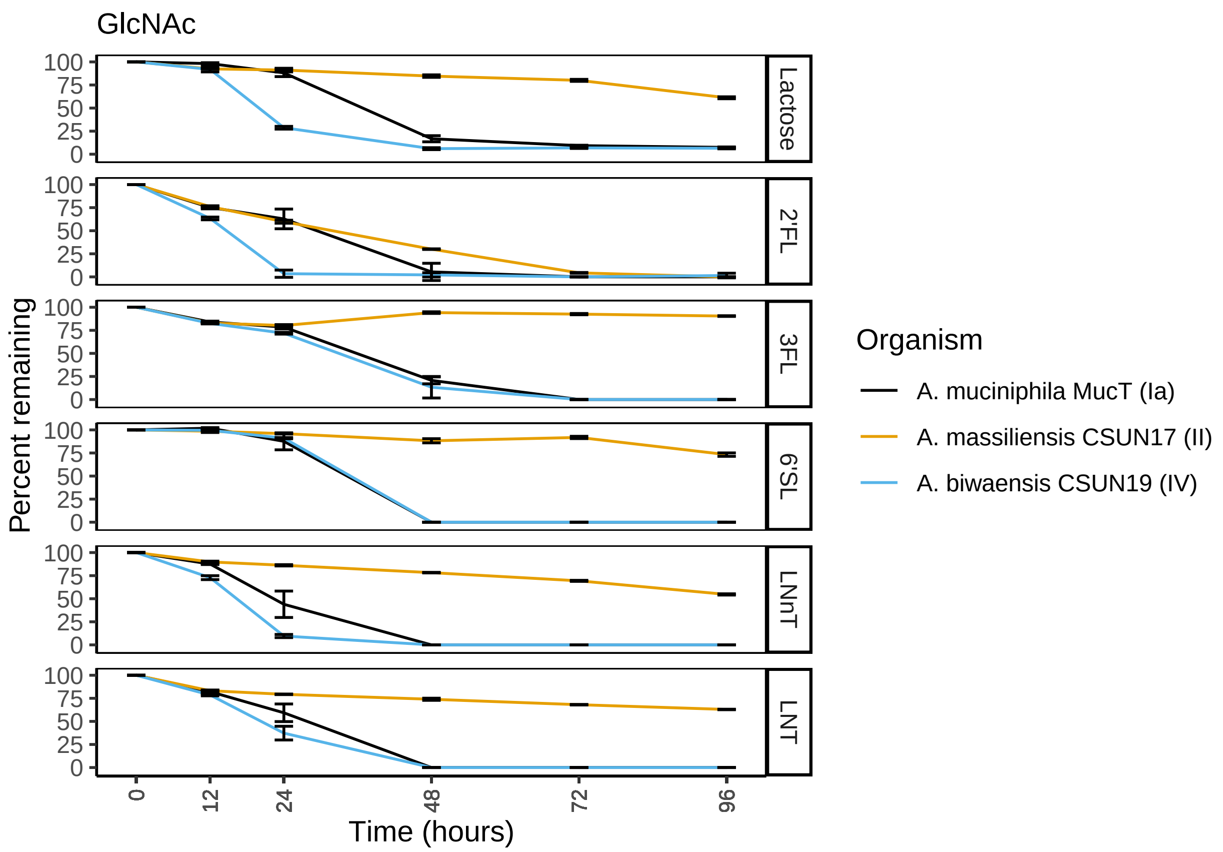


```
#caption=paste("made using", script.name, "\ngenerated on", Sys.time(), sep=" "))

#ggsave(here("Output","Figures",paste("iHMO_Timecourse_HMOs",format(Sys.time(), "%Y-%m-%d-%H-%M"),"png"
```

##Graph Sugar data

```
Sugars.stats.2 <- Sugars.stats
Sugars.stats.2$Org <- gsub("MucT", "A. muciniphila MucT (Ia)",gsub("CSUN-17", "A. massiliensis CSUN17 (I
Sugars.stats.2$Org <- factor(Sugars.stats.2$Org, levels=c("A. muciniphila MucT (Ia)", "A. massiliensis C
```

```
Sugars.stats.2$HMO_f <- factor(Sugars.stats.2$HMO, levels=c("Lactose", "2'FL", "3FL", "6'SL", "LNnT", "I

Sialic.stats <- Sugars.stats.2[grepl("Sialic",Sugars.stats.2$Name),] #Get just sialic acid
Fucose.stats <- Sugars.stats.2[grepl("Fucose",Sugars.stats.2$Name),] #Get just fucose

#Create a data frame of empty data so that it also plots CSUN-17 (that has no fucose)
Fucose.stats.2 <-data.frame("Fucose", "3FL", "A. massiliensis CSUN17 (II)", "BTTM + GlcNAc
", 0, 3, NA, NA, "3FL")
names(Fucose.stats.2)<-c("Name", "HMO", "Org", "Base", "Time", "N", "Average", "SD", "HMO_f"  )
Fucose.stats.3 <- rbind(Fucose.stats, Fucose.stats.2)
NAG.stats <- Sugars.stats.2[grepl("N-acetyl",Sugars.stats.2$Name),] #Get just NAG


#Plot SIALIC ACID graph
Sialic.stats %>%
  ggplot( aes(Time, Average, group=Org)) +
  geom_col(aes(fill=Org), na.rm = TRUE, position = "dodge") + #bar graph
  geom_errorbar(aes(ymin = Average - ifelse(Average < 0, SD, 0),
        ymax = Average + ifelse(Average > 0, SD, 0)), position="dodge") + #unidirectional errorbar
  facet_grid(HMO_f~Org, scales="free_x", space="free_x", labeller = label_wrap_gen(width=5)) +  #create
  theme_classic() + #get rid of the grey background
  theme(axis.text.x = element_text(angle = 90, vjust = 0.5, hjust=1, size = 8),
        panel.border = element_rect(color="black", fill=NA),
        strip.background =element_rect(fill="white"),
        strip.text.x = ggtext::element_textbox_simple(width = unit(1, "npc"),
                                                       height = unit(1, "lines"),
                                                       colour = 'black',
                                                       size = 7,
                                                       hjust = 0.5,
                                                       vjust = 0.5,
                                                       halign = 0.5,
                                                       valign = 0.5)) +
  scale_fill_manual(values = cbbPalette, name = "Organism") + #set plot colors
  scale_color_manual(values = cbbPalette, name = "Organism") + #set plot colors
  labs(x= "Time (hours)", y = "Concentration (mM)", title=NULL, subtitle = "GlcNAc",
       caption=NULL)+
  #caption=paste("made using", script.name, "\ngenerated on", Sys.time(), sep=" "))+
  ylim(0,4)
```

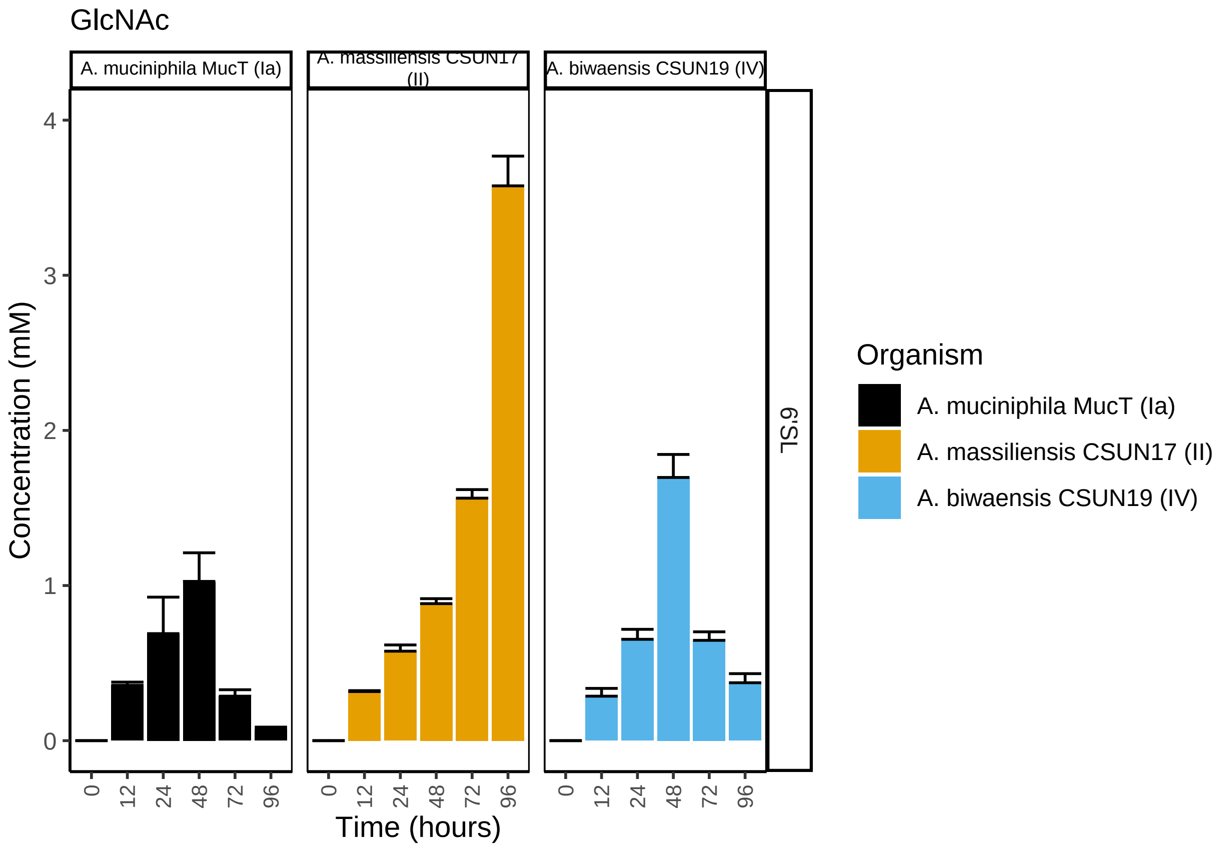


```
ggsave(here("Output","Figures",paste("iHMO_Timecourse_Sialic",format(Sys.time(), "%Y-%m-%d-%H-%M"),"png

#Plot FUCOSE graph
Fucose.stats.3 %>%
  ggplot( aes(Time, Average, group=Org)) +
  geom_col(aes(fill=Org), na.rm = TRUE, position = "dodge") + #bar graph
  geom_errorbar(aes(ymin = Average - ifelse(Average < 0, SD, 0),
        ymax = Average + ifelse(Average > 0, SD, 0)), position="dodge") + #unidirectional errorbar
  facet_grid(HMO_f~Org, scales="fixed", space="free") +  #create sub-plots
  theme_classic() + #get rid of the grey background
  theme(axis.text.x = element_text(angle = 90, vjust = 0.5, hjust=1, size = 8),
        panel.border = element_rect(color="black", fill=NA),
        strip.background =element_rect(fill="white"),
        strip.text.x = ggtext::element_textbox_simple(width = unit(1, "npc"),
                                                     height = unit(1, "lines"),
                                                     colour = 'black',
                                                     size = 7,
                                                     hjust = 0.5,
                                                     vjust = 0.5,
                                                     halign = 0.5,
                                                     valign = 0.5)) +
  scale_fill_manual(values = cbbPalette, name = "Organism") + #set plot colors
  scale_color_manual(values = cbbPalette, name = "Organism") + #set plot colors
  labs(x= "Time (hours)", y = "Concentration (mM)", title=NULL, subtitle = "GlcNAc",
      caption=NULL)+
  #caption=paste("made using", script.name, "\ngenerated on", Sys.time(), sep=" ")) +
```

```
  ylim(0,1.1)
```

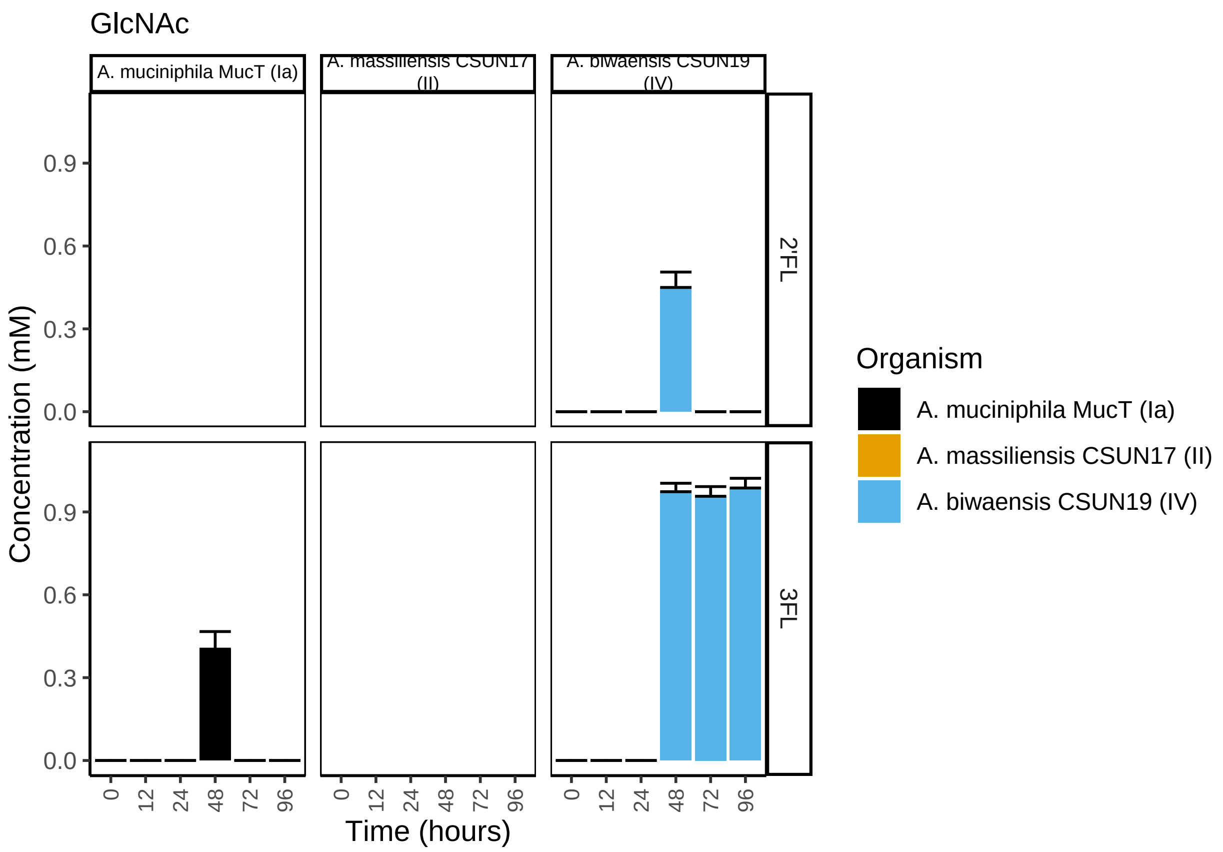


```
#ggsave(here("Output","Figures",paste("iHMO_Timecourse_Fucose",format(Sys.time(), "%Y-%m-%d-%H-%M"),"pn
```

Graph Lactose data

```
Lactose.stats <- Sugars.stats[grepl("Lactose",Sugars.stats$Name),] %>%
    mutate(Time=as.numeric(Time))
Lactose.stats$Org <- gsub("MucT", "A. muciniphila MucT (Ia)",gsub("CSUN-17", "A. massiliensis CSUN17 (I
Lactose.stats$Org <- factor(Lactose.stats$Org, levels=c("A. muciniphila MucT (Ia)", "A. massiliensis CSU
Lactose.stats$HMO <- factor(Lactose.stats$HMO, levels=c("Glucose", "Lactose", "2'FL", "3FL", "6'SL", "LN

Lactose.stats %>%
  filter(HMO!="Glucose" & HMO!="Lactose")%>%
  filter(HMO!="LNnT" & HMO!="LNT") %>%
  ggplot( aes(Time, Average, group=Org)) +
  geom_line(aes(color=Org), na.rm = TRUE) + #bar graph
  geom_errorbar(aes(ymin = Average - SD,
                    ymax = Average + SD), width=3) + #unidirectional errorbar
  facet_grid(HMO~., scales="free_x", space="free_x") +  #create sub-plots
  theme_classic() + #get rid of the grey background
  theme(axis.text.x = element_text(angle = 90, vjust = 0.5, hjust=1, size = 8),
        panel.border = element_rect(color="black", fill=NA),
```

```
       strip.background =element_rect(fill="white"),
       strip.text.x = ggtext::element_textbox_simple(width = unit(1, "npc"),
                                                    height = unit(1, "lines"),
                                                    colour = 'black',
                                                    size = 7,
                                                    hjust = 0.5,
                                                    vjust = 0.5,
                                                    halign = 0.5,
                                                    valign = 0.5)) +
  scale_color_manual(values = cbbPalette, name = "Organism") + #set plot colors
  scale_x_continuous(breaks = HMO.stats$Time, labels = HMO.stats$Time) +
  labs(x= "Time (hours)", y = "Concentration (mM)", title=NULL, subtitle = "GlcNAc",
       caption=NULL)+
  #caption=paste("made using", script.name, "\ngenerated on", Sys.time(), sep=" "))+
  ylim(-0.5,5)
```

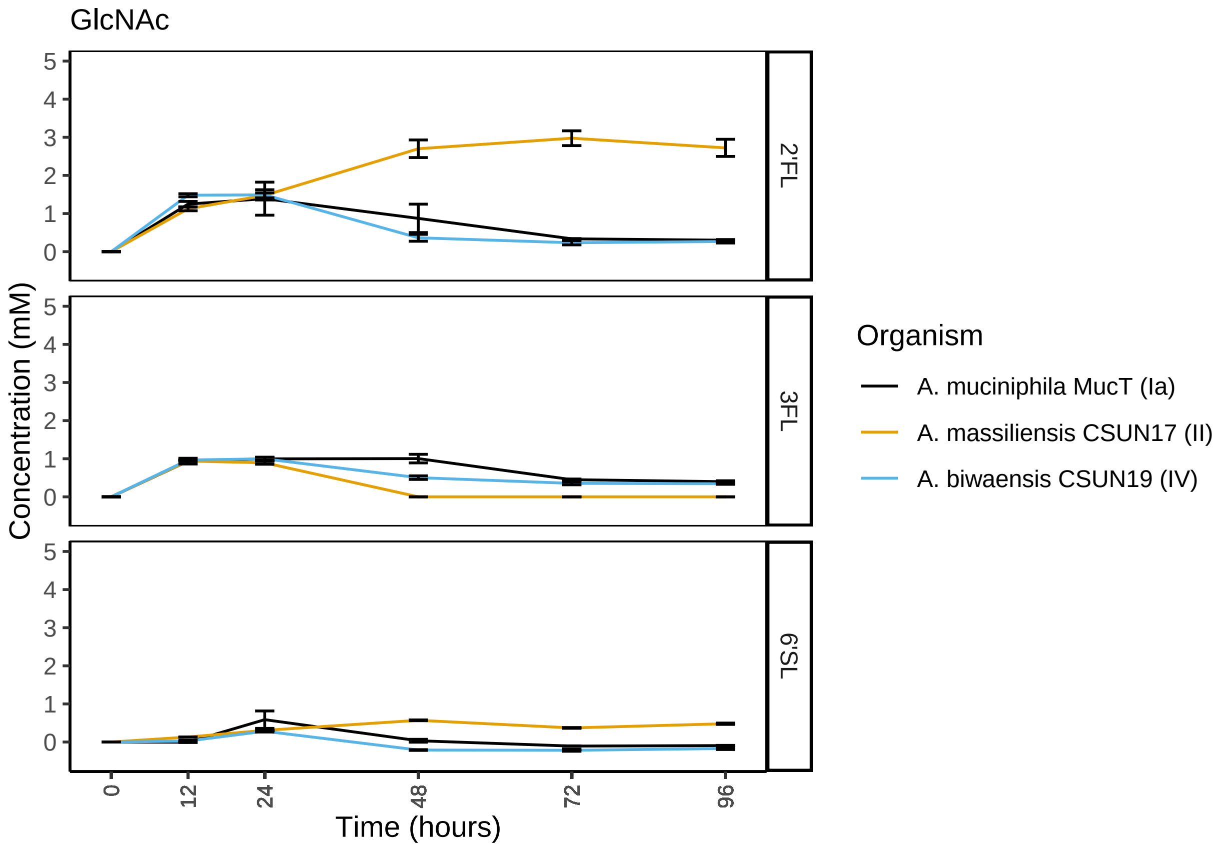


```
#ggsave(here("Output","Figures",paste("iHMO_Timecourse_Lactose",format(Sys.time(), "%Y-%m-%d-%H-%M"),"p
```

Graph GlcNAc data

```
Nacetyl.stats.2 <- Nacetyl.stats[grepl("N-acetyl-D-glucosamine",Nacetyl.stats$Name),]
Nacetyl.stats.2$Org <- gsub("MucT", "A. muciniphila MucT (Ia)",gsub("CSUN-17", "A. massiliensis CSUN17
Nacetyl.stats.2$Org <- factor(Nacetyl.stats.2$Org, levels=c("A. muciniphila MucT (Ia)", "A. massiliensis
```

```r
Nacetyl.stats.2$HMO <- factor(Nacetyl.stats.2$HMO, levels=c("Glucose", "Lactose", "2'FL", "3FL", "6'SL"

Nacetyl.stats.2 %>%
#  filter(HMO!="Glucose" & HMO!="Lactose")%>%
#  filter(HMO!="LNnT" & HMO!="LNT") %>%
  ggplot( aes(Time, Average, group=Org)) +
  geom_col(aes(fill=Org), na.rm = TRUE, position = "dodge") + #bar graph
  geom_errorbar(aes(ymin = Average - ifelse(Average < 0, SD, 0),
        ymax = Average + ifelse(Average > 0, SD, 0)), position="dodge") + #unidirectional errorbar
  facet_grid(HMO~Org, scales="fixed", space="free") +  #create sub-plots
  theme_classic() + #get rid of the grey background
  theme(axis.text.x = element_text(angle = 90, vjust = 0.5, hjust=1, size = 8),
        panel.border = element_rect(color="black", fill=NA),
        strip.background =element_rect(fill="white"),
        strip.text.x = ggtext::element_textbox_simple(width = unit(1, "npc"),
                                                      height = unit(1, "lines"),
                                                      colour = 'black',
                                                      size = 7,
                                                      hjust = 0.5,
                                                      vjust = 0.5,
                                                      halign = 0.5,
                                                      valign = 0.5)) +
  scale_fill_manual(values = cbbPalette, name = "Organism") + #set plot colors
  scale_color_manual(values = cbbPalette, name = "Organism") + #set plot colors
  labs(x= "Time (hours)", y = "Concentration (mM)", title=NULL, subtitle = "GlcNAc",
       caption=NULL)+
  #caption=paste("made using", script.name, "\ngenerated on", Sys.time(), sep=" ")) +
    ylim(-0.5,10)
```

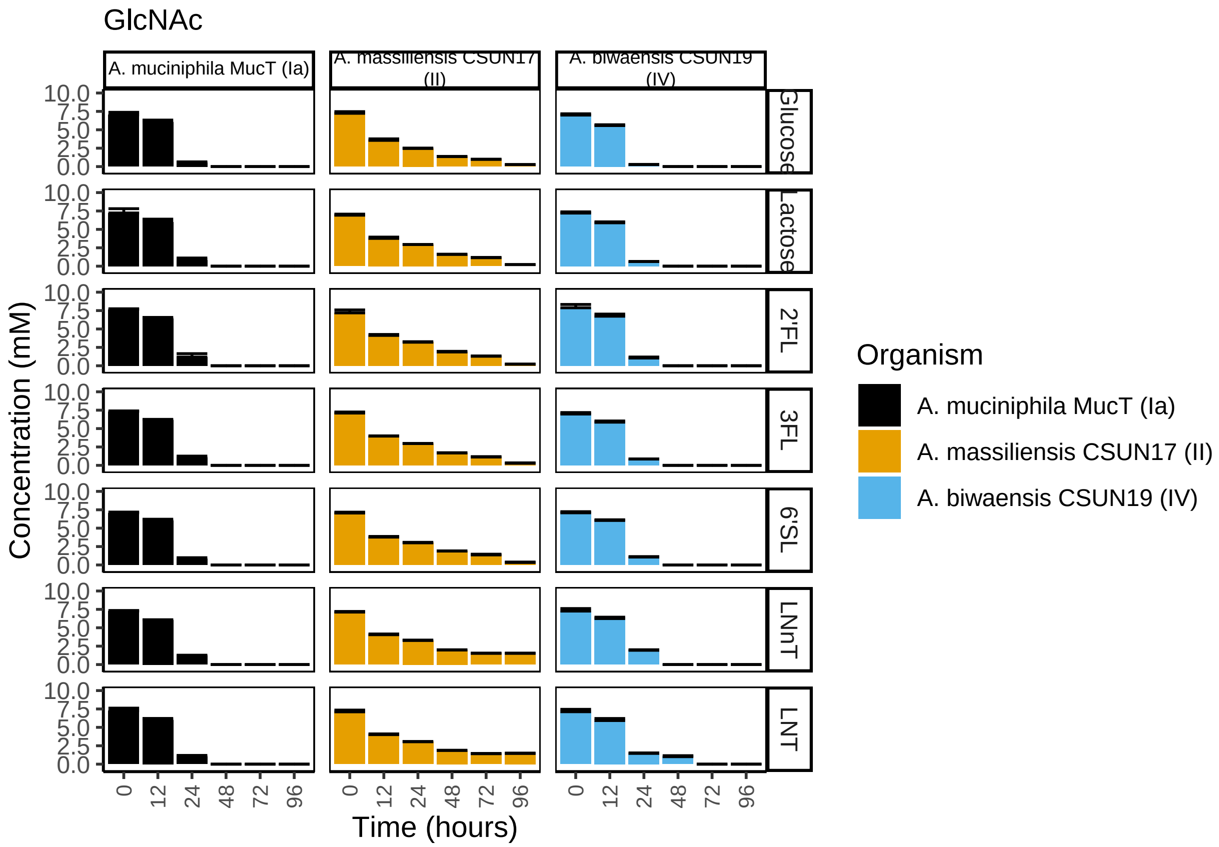


```
ggsave(here("Output","Figures",paste("iHMO_Timecourse_GlcNAc_bar",format(Sys.time(), "%Y-%m-%d-%H-%M"),
```

Graph Glucose data

```
Sugars.stats.2 <- Sugars.stats %>% mutate(Time=as.numeric(Time))
Sugars.stats.2$Org <- gsub("MucT", "A. muciniphila MucT (Ia)",gsub("CSUN-17", "A. massiliensis CSUN17 (I
Sugars.stats.2$Org <- factor(Sugars.stats.2$Org, levels=c("A. muciniphila MucT (Ia)", "A. massiliensis C

Glucose.stats <- Sugars.stats.2[grepl("Glucose",Sugars.stats.2$Name),] %>%
    mutate(Time=as.numeric(Time))

Glucose.stats %>%
  ggplot( aes(Time, Average, group=Org)) +
  geom_line(aes(color=Org), na.rm = TRUE) + #bar graph
  geom_errorbar(aes(ymin = Average - SD,
                    ymax = Average + SD), width=3) + #unidirectional errorbar
  facet_grid(HMO~., scales="free_x", space="free_x") +  #create sub-plots
  theme_classic() + #get rid of the grey background
  theme(axis.text.x = element_text(angle = 90, vjust = 0.5, hjust=1, size = 8),
        panel.border = element_rect(color="black", fill=NA),
        strip.background =element_rect(fill="white"),
        strip.text.x = ggtext::element_textbox_simple(width = unit(1, "npc"),
                                                       height = unit(1, "lines"),
```

```
                                         colour = 'black',
                                         size = 7,
                                         hjust = 0.5,
                                         vjust = 0.5,
                                         halign = 0.5,
                                         valign = 0.5)) +
  scale_color_manual(values = cbbPalette, name = "Organism") + #set plot colors
  scale_x_continuous(breaks = HMO.stats$Time, labels = HMO.stats$Time) +
  labs(x= "Time (hours)", y = "Concentration (mM)", title=NULL, subtitle = "GlcNAc",
       caption=NULL)
```

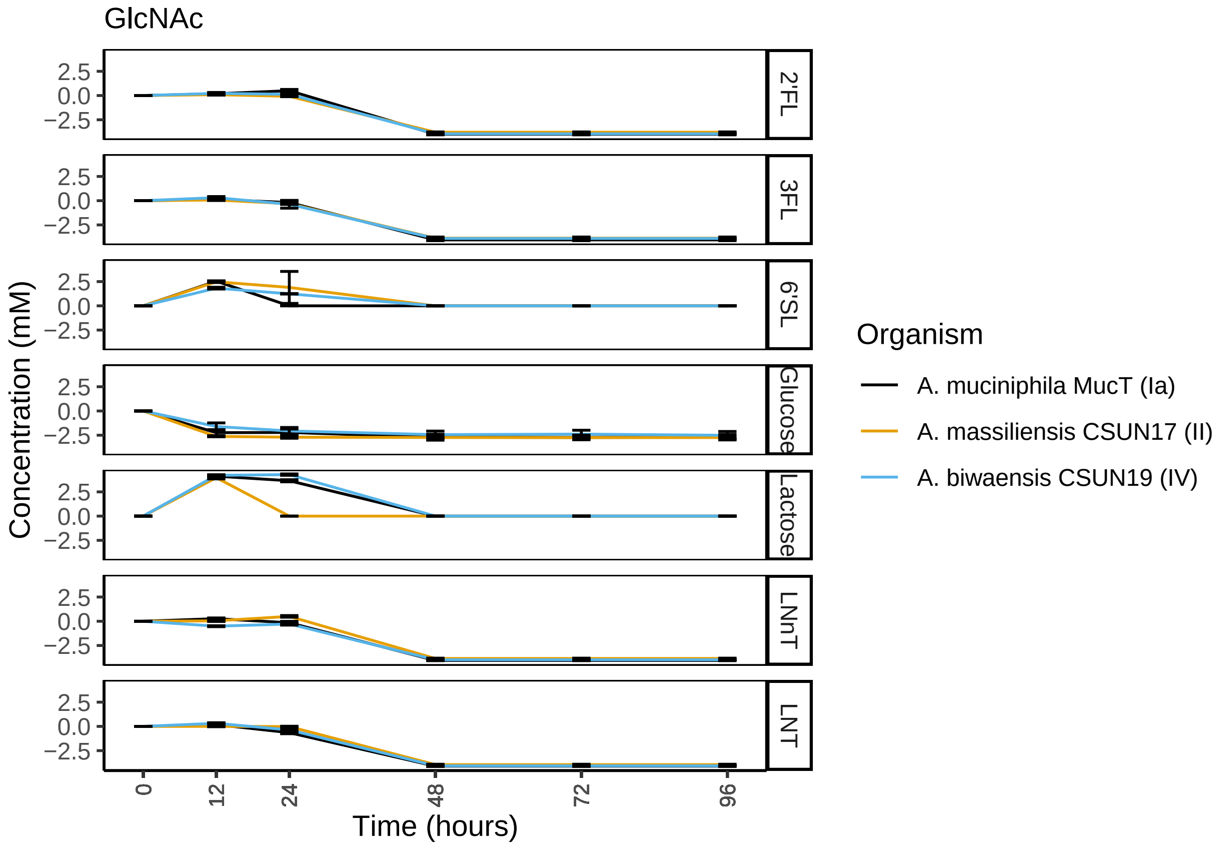


```
#caption=paste("made using", script.name, "\ngenerated on", Sys.time(), sep=" "))

#ggsave(here("Output","Figures",paste("iHMO_Timecourse_Glucose",format(Sys.time(), "%Y-%m-%d-%H-%M"),"p
```

#STATS ## Statistical Tests on the data

```
##Test if the data is normally distributed (shaprio test) p>0.05 = normal distribution (significance = 
HMO.clean.samples %>%
  group_by(Name) %>%
  shapiro_test(Concent)
```

```
## # A tibble: 6 x 4
##   Name     variable statistic             p
```

```
##   <chr>   <chr>        <dbl>         <dbl>
## 1 2'-FL   Concent      0.811 0.000000744
## 2 3'-FL   Concent      0.772 0.0000000937
## 3 6'-SL   Concent      0.699 0.00000000323
## 4 LNT     Concent      0.826 0.00000175
## 5 LNnt    Concent      0.817 0.00000105
## 6 Lactose Concent      0.818 0.00000111
```

```r
##Quick histogram to visualize non-normality
HMO.clean.samples %>%
ggplot( aes(x=Concent, color=Name, fill=Name)) +
    geom_histogram(alpha=0.6, binwidth = 5) +
  facet_wrap(~Name)
```

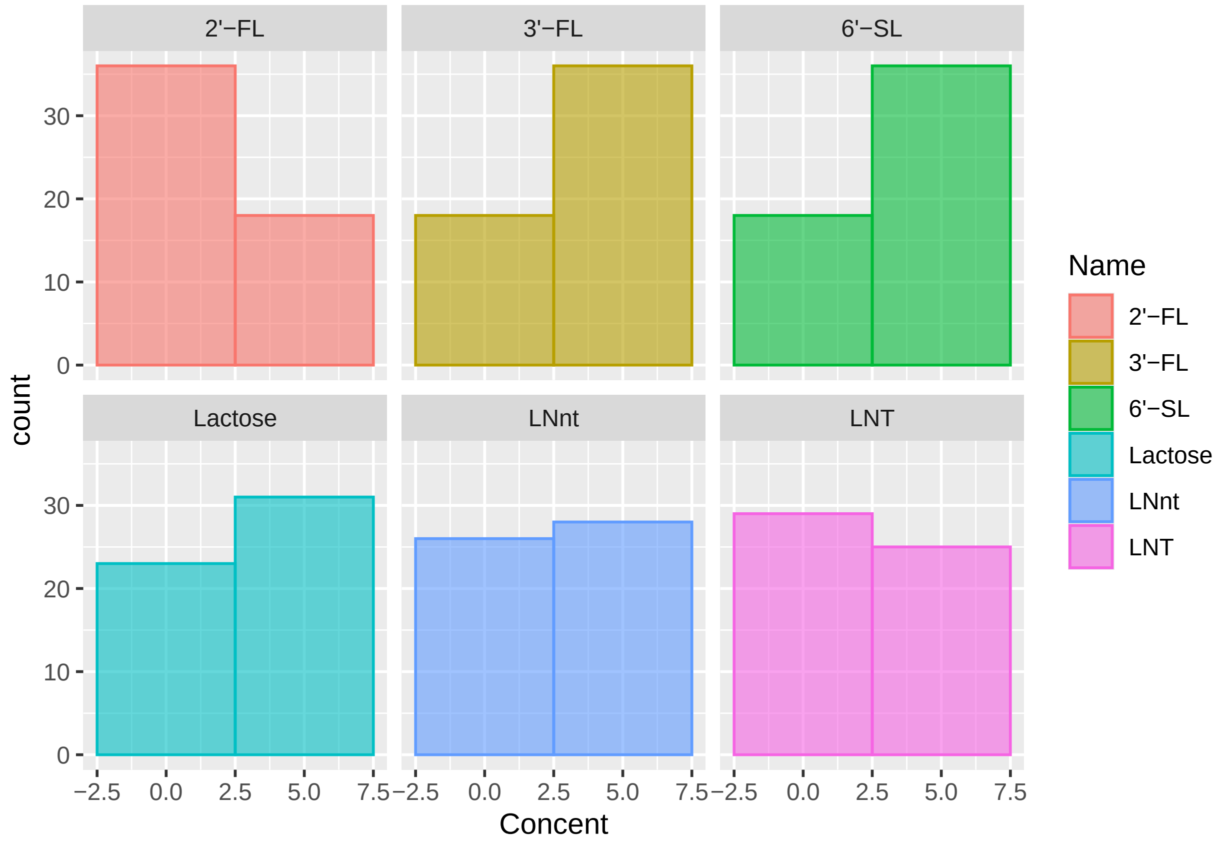


```r
##Calculate statistical significance (pvalues)
##SCFA
SCFA.kw.test <- SCFA.clean.samples %>%
  filter(Time == "96") %>%
  filter(Name != "1,2-propanediol") %>%
  group_by(Name, HMO) %>%
  kruskal_test(Concent ~ Org)

SCFA.dunn.test <- SCFA.clean.samples %>%
  filter(Time == "96") %>%
  filter(Name != "1,2-propanediol") %>%
```

```r
  group_by(Name, HMO) %>%
  dunn_test(Concent ~ Org) %>%
  adjust_pvalue(method = "bonferroni")


##Calculate statistical significance (pvalues)
##HMO
HMO.kw.test <- HMO.clean.samples %>%
  filter(Time == "96") %>%
  filter(is.finite(ConcSubtr)) %>%
  group_by(Name, HMO) %>%
  kruskal_test(ConcSubtr ~ Org)

HMO.dunn.test <- HMO.clean.samples %>%
  filter(Time == "96") %>%
  group_by(Name) %>%
  dunn_test(ConcSubtr ~ Org) %>%
  adjust_pvalue(method = "bonferroni") %>%
  mutate(HMO = paste(Name))

##Calculate statistical significance (pvalues)
##Sugars
Sugar.kw.test <- Sugars.clean.samples %>%
  filter(Time == "96") %>%
  filter(HMO != "LNT" & HMO != "LNnT" & HMO != "Glucose") %>%
  filter(Name != "Fucose" & Name != "Glucose" & Name != "N-acetyl-D-glucosamine") %>%
  group_by(Name, HMO) %>%
  kruskal_test(Concent ~ Org)

Sugar.dunn.test <- Sugars.clean.samples %>%
  filter(Time == "96") %>%
  filter(HMO != "LNT" & HMO != "LNnT" & HMO != "Glucose") %>%
  filter(Name != "Fucose" & Name != "Glucose" & Name != "N-acetyl-D-glucosamine") %>%
  group_by(Name, HMO) %>%
  dunn_test(Concent ~ Org) %>%
  adjust_pvalue(method = "bonferroni")


##MERGE data frames
KW.list <- list(SCFA.kw.test, HMO.kw.test, Sugar.kw.test)
KW.merge <- data.frame(do.call(rbind, KW.list))

Dunn.list <- list(SCFA.dunn.test, HMO.dunn.test, Sugar.dunn.test)
Dunn.merge <- data.frame(do.call(rbind, Dunn.list))
```
