# Supplementary material for: Time-resolved growth of diverse human-associated Akkermansia on human milk oligosaccharides
Source: Microbiol Spectr. 2026 Jan 27;14(3):e02071-25. doi: 10.1128/spectrum.02071-25 (PMC12955465; doi:10.1128/spectrum.02071-25)
Supplement: File S3 — R scripts used to generate figures and statistics. [file spectrum.02071-25-s0003.pdf]

SpectroMaxGraphing

Ashwana

02/20/2023

Script Details:

- This script takes the output from the nanostar plate reader and a plate map and automates graphing *Input file 1: growth curve csv file from the plate reader* Input file 2: plate layout generated in excel and saved as a csv *Output file: png, pdf, or tif file of the growth curve

Load packages

may have to install them first

to install, go to "Tools" > "Install Packages…" and type in the corresponding package name

```
library(reshape2) #melt
library(dplyr) #average
```

```
##
## Attaching package: 'dplyr'

## The following objects are masked from 'package:stats':
##
##     filter, lag

## The following objects are masked from 'package:base':
##
##     intersect, setdiff, setequal, union
```

```
library(ggplot2) #plot
library(stringr) #add zeroes
library(tidyr) #separate
```

```
##
## Attaching package: 'tidyr'

## The following object is masked from 'package:reshape2':
##
##     smiths
```

```r
library(here) #set working directory
```

```
## here() starts at /Users/africker/Desktop/DrAsh/02 Data/18 iHMO
```

```r
library(purrr) #map_df
```

Load files

Input files needed:

- .csv file from plate reader. Count the number of lines before it reaches the word "Time". Skip that number of lines.
- .csv file created by the user for the plate layout

```r
files <- list.files(here("VitaminB12", "Input"), pattern = "C02.csv", full.names = TRUE) #Finds all gro
#Read in growth curve file
dd.od <- lapply(1:length(files), function(idx) {
    # load the file
    d <-read.csv(files[idx], header = TRUE, sep=",", check.names = FALSE)
    # now melt immediately
    d.m <- melt(d, c("Well"), c(1:length(d)))
}) #iterates over file list and reads them all in as CSV

dd.od <- setNames(dd.od, str_match(list.files(here("VitaminB12", "Input"), pattern="Clrbtm.csv"), "0\\s
#Searches the name of each file for the term "AH_" and the final ".csv" and captures everything in betw

ODs <- map_df(dd.od, ~as.data.frame(.x), .id="Plate") #Merge the data together
ODs$Well <- paste0(ODs$Well, ODs$variable, sep="") #make the Well column a true well value column

#Read in plate map file
PM <- read.csv(here("VitaminB12","Input","DrAsh_VitaminB12_In_Mucin_Trial_6_28_24_AH_PlateMap.csv"),
                     header=T, #The file does not have headers (false)
                     sep=',', #The file is a comma separated file, so the separator is comma
                     check.names = F) #Not really necessary, but allows us to have spaces in the header
```

Format files to be merged together

```r
##Reformat the growth curve (GC) file to work with the plate map (PM)
ODs <- ODs[ , colSums(is.na(ODs))==0] #remove columns that have no data
ODs <- ODs[!grepl("Well", ODs$variable),] #remove columns that have no data
ODs$value <- as.numeric(ODs$value) #make the value column numeric

#Convert to OD600
#The conversions are in 01 Protocols/02 Bacterial Cultures/SpectroStarNano_to_Cuvette_AH_Dec2022.xslx
normOD <- ODs #copy the data frame to normalize (keep original data frame for optional modifications, b

max_open_cuv <- function(x){return ((2.0562*x)-0.0962)} #function to convert SPECTROMAX to cuvette OD60
normOD$OD600 <-sapply(normOD$value,max_open_cuv) #apply function to the spectramax values
```

```
##Reformats the plate map (PM) file to work with the growth curve (GC)
reshapPM <- melt(PM, id="Well",
     value.name="Sample") #Reshape the data frame into linear format
reshapPM$Well <- paste(reshapPM$Well, reshapPM$variable, sep='') #add a 'Well' column with the correspo

##The next command combines the two reshaped files
combOD <- as.data.frame(merge(x=normOD, y=reshapPM, by=c("Well","variable"))[])
```

Reshape file and calculate averages

```
#This command calculates the averages and standard deviation
statsOD <- combOD %>%
              group_by(Sample, Plate) %>%
              summarise(N=length(OD600),
                        Average=mean(OD600),
                        SD=sd(OD600))
```

```
## `summarise()` has grouped output by 'Sample'. You can override using the
## `.groups` argument.
```

```
#Decide which samples you want to plot. If you want to plot everything, can skip to next code chunk
unique(combOD$Sample) #Print out all unique sample types.
```

```
##  [1] "2.5 ng/mL + Blank + None"  "2.0 ng/mL + Blank + None"
##  [3] "1.5 ng/mL + Blank + None"  "1.0 ng/mL + Blank + None"
##  [5] "0 ng/mL + Blank + None"    "Old Mucin + Blank + None"
##  [7] "New Mucin + Blank + None"  "empty"
##  [9] "2.5 ng/mL + PBS + RT"      "2.0 ng/mL + PBS + RT"
## [11] "1.5 ng/mL + PBS + RT"      "1.0 ng/mL + PBS + RT"
## [13] "0 ng/mL + PBS + RT"        "Old Mucin + PBS + RT"
## [15] "New Mucin + PBS + RT"      "2.5 ng/mL + Water + RT"
## [17] "2.0 ng/mL + Water + RT"    "1.5 ng/mL + Water + RT"
## [19] "1.0 ng/mL + Water + RT"    "0 ng/mL + Water + RT"
## [21] "Old Mucin + Water + RT"    "New Mucin + Water + RT"
## [23] "2.5 ng/mL + PBS + 4degC"   "2.0 ng/mL + PBS + 4degC"
## [25] "1.5 ng/mL + PBS + 4degC"   "1.0 ng/mL + PBS + 4degC"
## [27] "0 ng/mL + PBS + 4degC"     "Old Mucin + PBS + 4degC"
## [29] "New Mucin + PBS + 4degC"   "2.5 ng/mL + Water + 4degC"
## [31] "2.0 ng/mL + Water + 4degC" "1.5 ng/mL + Water + 4degC"
## [33] "1.0 ng/mL + Water + 4degC" "0 ng/mL + Water + 4degC"
## [35] "Old Mucin + Water + 4degC" "New Mucin + Water + 4degC"
```

Graph the data PART 1

```
combOD2 <- separate(statsOD, Sample, into = c("Concentration" ,"Type"), sep="\\s+", remove = FALSE) #sp

#Creates one plot with the specified values
ggplot(combOD2, aes(Sample, Average, group=Sample)) + #can choose data to plot (all=statsSD, GCSubset, 
```

```
  geom_col(fill="black") + #Make it a bar graph
  geom_errorbar(aes(ymin=Average-SD, ymax=Average+SD), colour = "black", width = 0.5) + #errorbars inst
  facet_grid(Plate~Type, space="free", scale="free")+
  labs(x="Sample", y="Absorbance at 600 nm", title = "Vitamin B12") + #Set axis and title labels
  theme_bw() + #Change background to a black and white
  theme(panel.grid = element_blank(), #Remove the x- and y- lines on the graph
        axis.text.x = element_text(angle=90, size=10)) + #Change format of x-axis
  scale_x_discrete(expand = c(0, 0)) #Start the graph at the y axis (otherwise it starts offset)
```

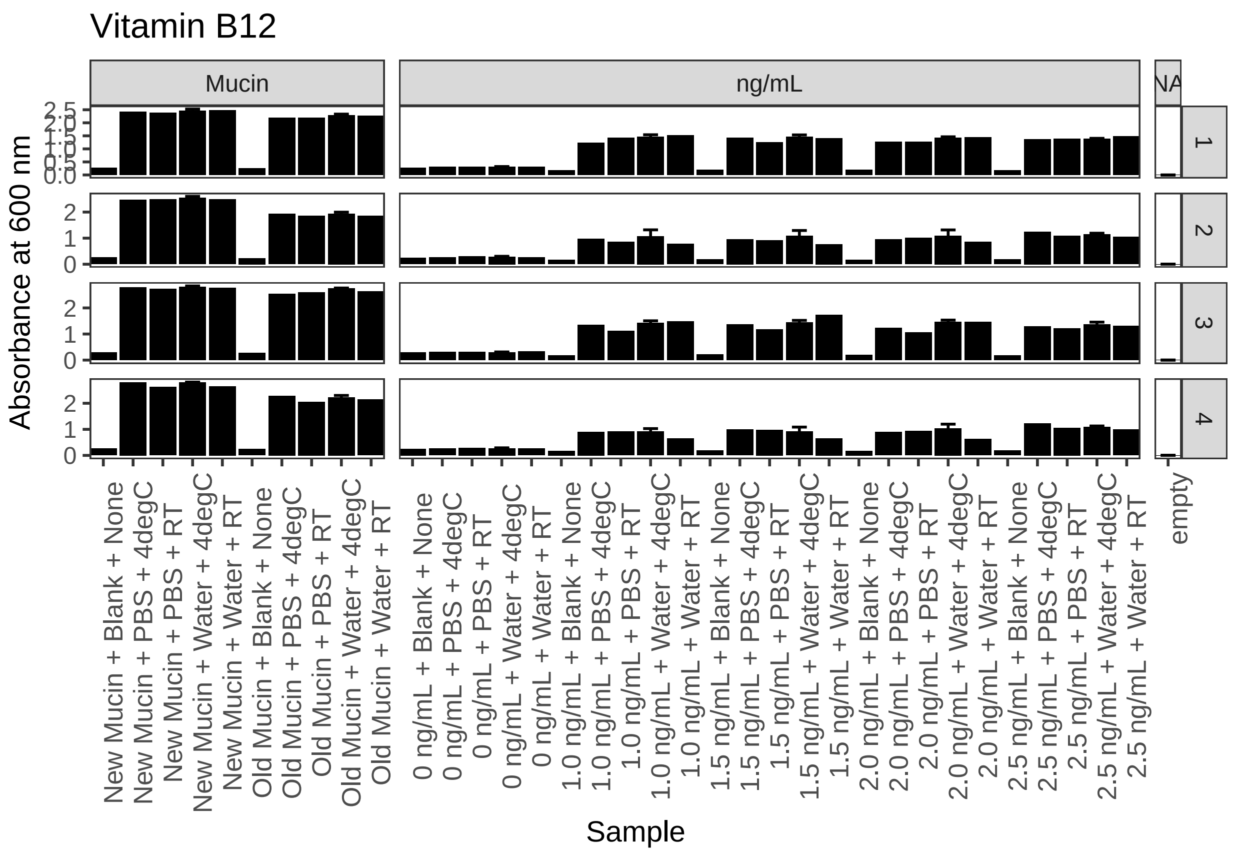


```
#ggsave(here("VitaminB12", "Output", paste(format(Sys.time(),"%m-%d-%Y-%H-%M"), "AllData.v3.png")), wid
```

Graph the data 2

```
#Use the previous code output to choose what you want to plot (optional)
ODSubset <- combOD[!grepl("empty|2.5 ng/mL|1.25 ng/mL|0.25 ng/mL|0.25%", combOD$Sample),] #subset sampl

statsODSubset <- ODSubset %>%
              group_by(Sample, Plate) %>%
              summarise(N=length(OD600),
                        Average=mean(OD600),
                        SD=sd(OD600))
```

```
## `summarise()` has grouped output by 'Sample'. You can override using the
```

```
## `.groups` argument.
```

```r
combODSubset <- separate(statsODSubset, Sample, into = c("Add" ,"Wash" , "Temp"), sep="\\+", remove = F
  mutate_if(is.character, str_trim) %>%
  filter(Plate==1 & Wash =="Water" & Temp=="4degC")
```

```
## `mutate_if()` ignored the following grouping variables:
## * Column `Sample`
```

```r
#Rename samples and set factors (optional)
ODSubset$Sample<-gsub("4degC","4C",ODSubset$Sample) #rename for fancy plotting
combODSubset$Add <- gsub("New Mucin", "Mucin Batch 1", gsub("Old Mucin", "Mucin Batch 2", combODSubset$
combODSubset$Add_f <- factor(combODSubset$Add, levels=c("0 ng/mL","1.0 ng/mL","1.5 ng/mL", "2.0 ng/mL",

#Creates one plot with the specified values
ggplot(combODSubset, aes(Add, Average, group=Sample)) + #can choose data to plot
  geom_col(fill="black") + #Make it a bar graph
  geom_errorbar(aes(ymin=Average-SD, ymax=Average+SD), colour = "black", width = 0.5) + #add errorbars
  labs(x=NULL, y="Absorbance at 600 nm", title = "Vitamin B12") + #Set axis and title labels
  theme_bw() + #Change background to a black and white
  theme(panel.grid = element_blank(), #Remove the x- and y- lines on the graph
        axis.text.x = element_text(angle=90, size=12, color="black"),
        axis.text.y = element_text(size=12, color="black"),
        axis.title.y = element_text(size=14, color="black"),
        title = element_text(size=14, color="black")) + #Change format of x-axis
  scale_x_discrete(expand = c(0, 0))+ #Start the graph at the y axis (otherwise it starts offset)
  scale_y_continuous(expand= c(0,0), limits = c(0,3), breaks=seq(0,3,by=1))
```

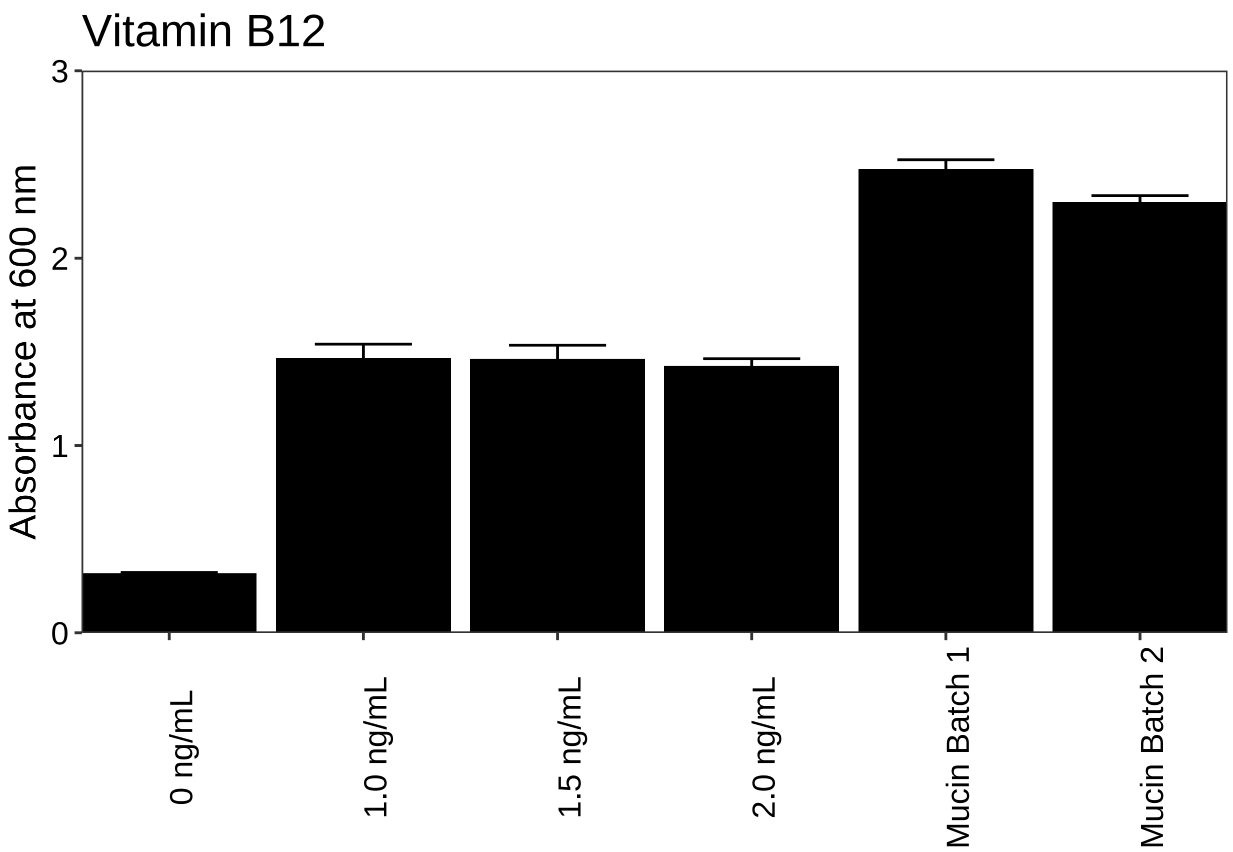


```
#ggsave(here("VitaminB12", "Output", paste("VitaminB12", unique(combODSubset$Plate), "v2.png", sep=".")),
```
