# Supplementary material for: Time-resolved growth of diverse human-associated Akkermansia on human milk oligosaccharides
Source: Microbiol Spectr. 2026 Jan 27;14(3):e02071-25. doi: 10.1128/spectrum.02071-25 (PMC12955465; doi:10.1128/spectrum.02071-25)
Supplement: Supplemental figure legends — Legends for Figures S1 to S5. [file spectrum.02071-25-s0004.docx]

**SUPPLEMENTAL FIGURE LEGENDS**

**Supplemental Figure 1.** Differences in growth yield are statistically significant across species, HMOs, and media. Maximum growth yield (A,B, C) and the time at which maximum growth was reached (D, E, F) was compared across species (A, D) across background media (B, E), and across HMOs (C, F). Representative strains used in this study are *A. muciniphila* MucT (Ia), *A. massiliensis* CSUN-17 (II), and *A. biwaensis* CSUN-19 (IV). Error bars represent standard deviation of four biological replicates ; statistical significance was determined by Dunn’s test with Bonferroni corrections following Kruskal-wallis in R. Symbol styles: (ns) non significant, (*) 0.05, (**) 0.01, (***) 0.001, (****) <0.001.

**Supplemental Figure 2.** Relative growth on HMOs is different across species. Growth on the mucin-only culture was subtracted from growth on an individual HMO at 24 hours (A) and 48 hours (B) for each species. Representative strains used in this study are *A. muciniphila* MucT (Ia), *A. massiliensis* CSUN-17 (II), and *A. biwaensis* CSUN-19 (IV). Error bars represent standard deviation of four biological replicates ; statistical significance was determined by Dunn’s test with Bonferroni corrections following Kruskal-wallis in R. Symbol styles: (ns) non significant, (*) 0.05, (**) 0.01, (***) 0.001, (****) <0.001.

**Supplemental Figure 3.** Despite presence of HMOs, in a mucin background, the doubling time and max slope for the first log phase are the same within a species. Representative strains used in this study are *A. muciniphila* MucT (Ia), *A. massiliensis* CSUN-17 (II), and *A. biwaensis* CSUN-19 (IV). Error bars represent standard deviation of three to four biological replicates ; red shading represents 95% confidence intervals ; statistical significance was determined by Dunn’s test with Bonferroni corrections following Kruskal-wallis in R. Symbol styles: (ns) non significant, (*) 0.05, (**) 0.01, (***) 0.001, (****) <0.001.

**Supplemental Figure 4.** Decreases in GlcNAc in all strains when grown in this background that is strain-dependent. Concentrations at each of 6 time points was determined by HPLC during growth on GlcNAc. Error bars represent standard deviation of three technical replicates.

**Supplemental Figure 5.** Mucin contains vitamin B12, supporting the growth of *Lactobacillus leichmanii*. Growth yield of *L. leichmanii* after cultivation with increasing concentrations of vitamin B12 or mucin. Error bars represent standard deviation of three technical replicates experiment was repeated two times and a representative figure is shown.

**Supplementary Table 1.** Carbon sources used in the experiment.

**Supplementary Table 2.** List of chemicals used for HMO and metabolite standard curves.
