# Supplementary figures and images for: Time-resolved growth of diverse human-associated Akkermansia on human milk oligosaccharides
Source: Microbiol Spectr. 2026 Jan 27;14(3):e02071-25. doi: 10.1128/spectrum.02071-25 (PMC12955465; doi:10.1128/spectrum.02071-25)

# Supp Figure 1

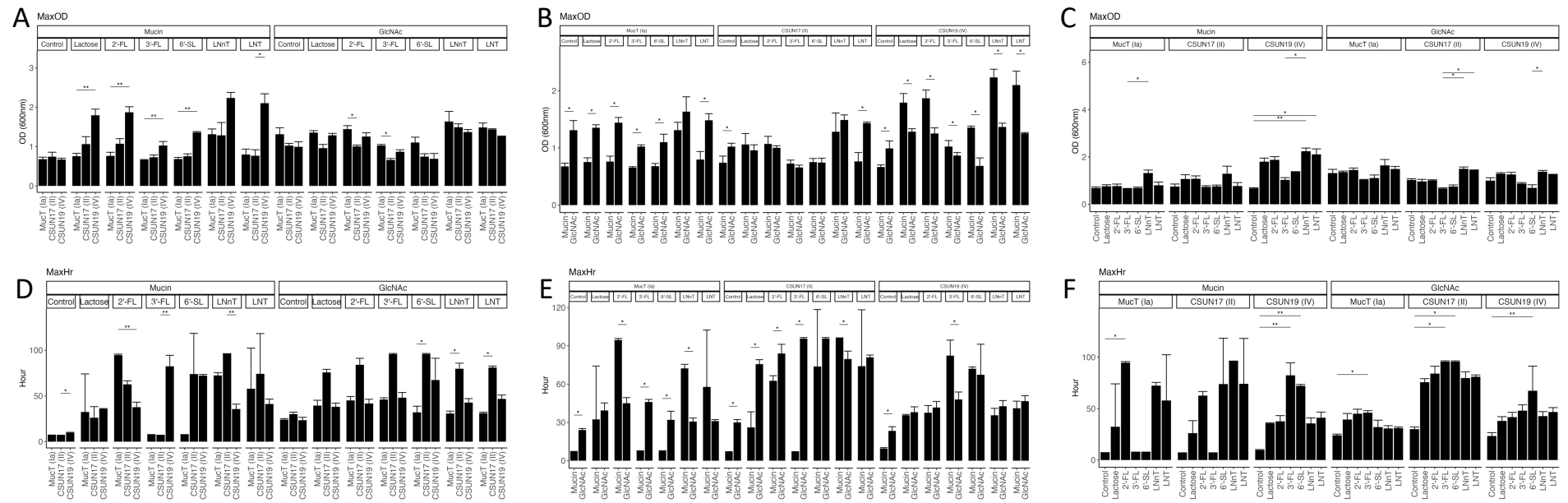

Supplement: Figure S1 — Differences in growth yield are statistically significant across species, HMOs, and media. [file spectrum.02071-25-s0005.pdf]

# Supp Figure 2

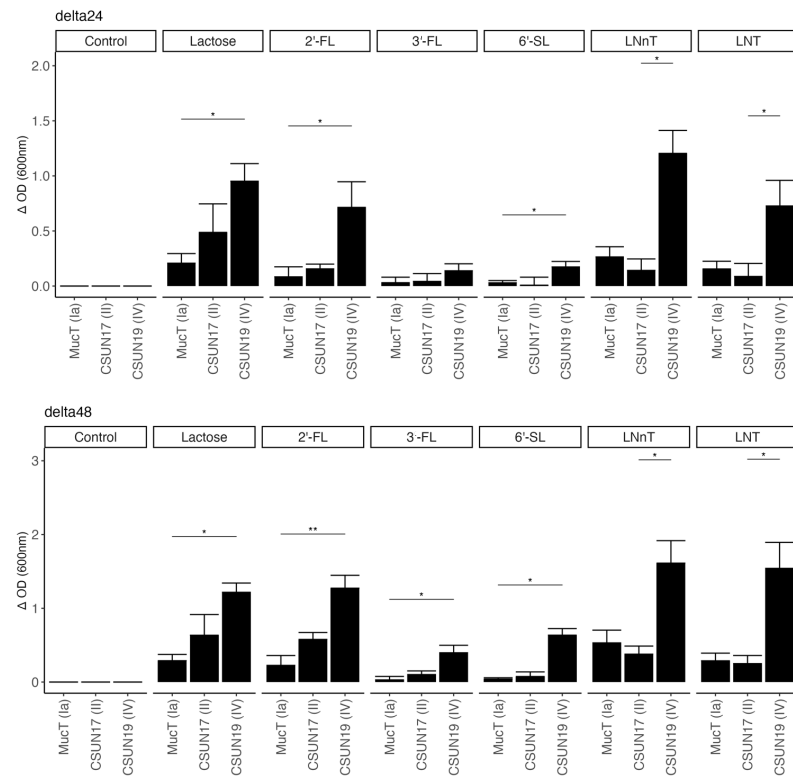

Supplement: Figure S2 — Relative growth on HMOs is different across species. Growth on the mucin-only culture was subtracted from growth on an individual HMO at 24 hours (A) and 48 hours (B) for each species. [file spectrum.02071-25-s0006.pdf]

# Supp Figure 3

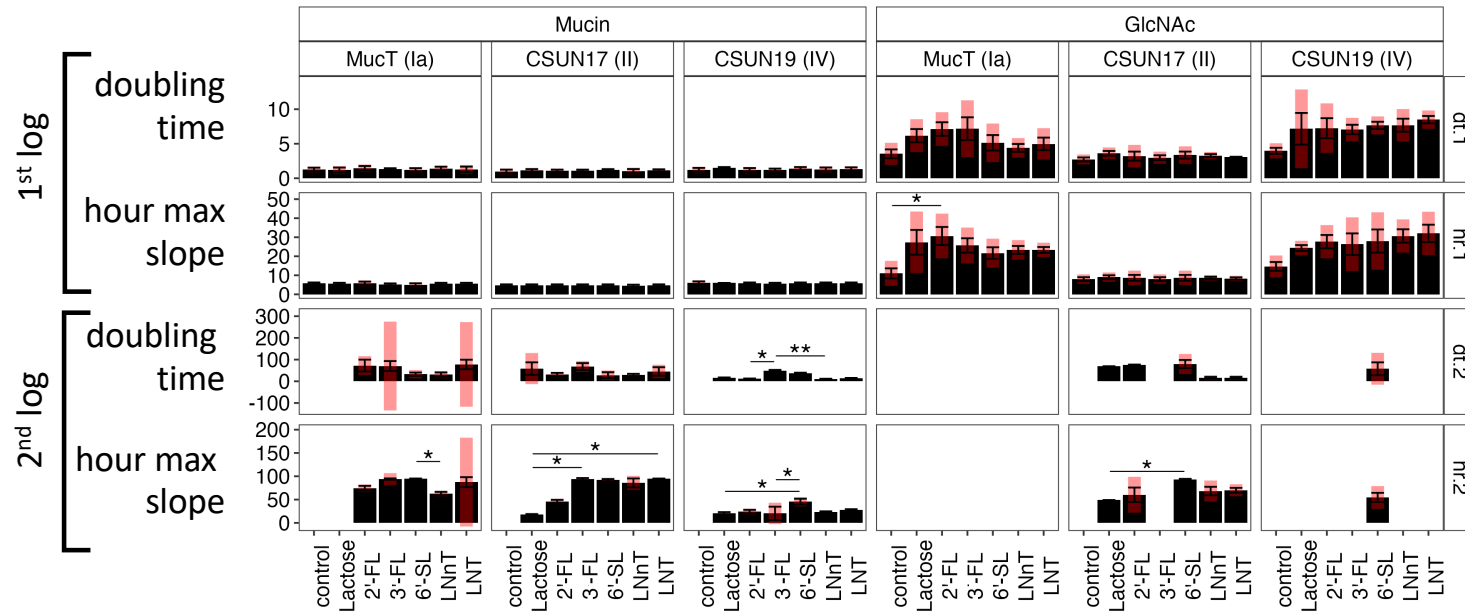

Supplement: Figure S3 — Despite the presence of HMOs, in a mucin background, the doubling time and max slope for the first log phase are the same within a species. [file spectrum.02071-25-s0007.pdf]

# Supp Figure 4

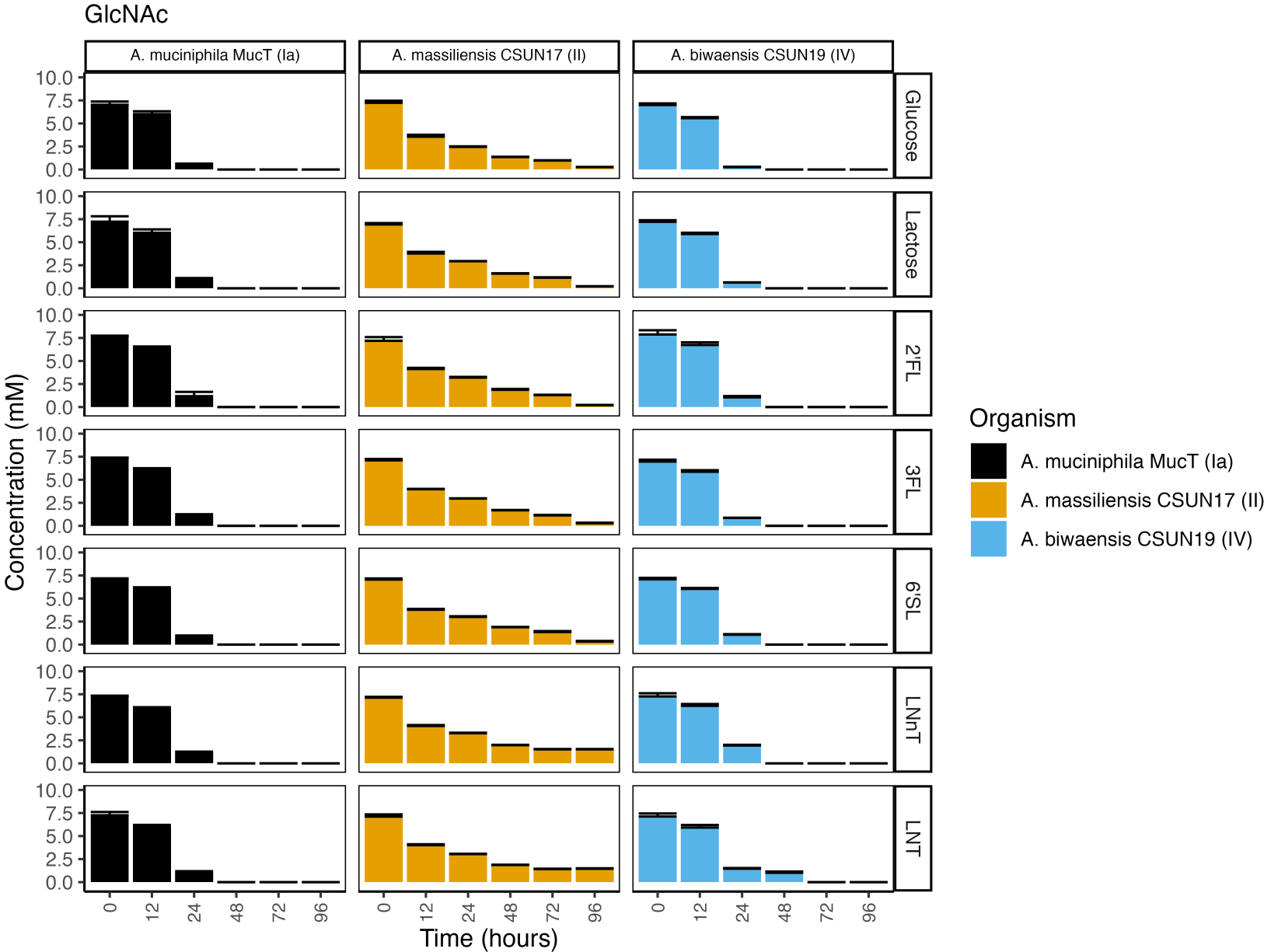

Supplement: Figure S4 — Decreases in GlcNAc in all strains when grown in this background that is rate-dependent. [file spectrum.02071-25-s0008.pdf]

# Supp Figure 5

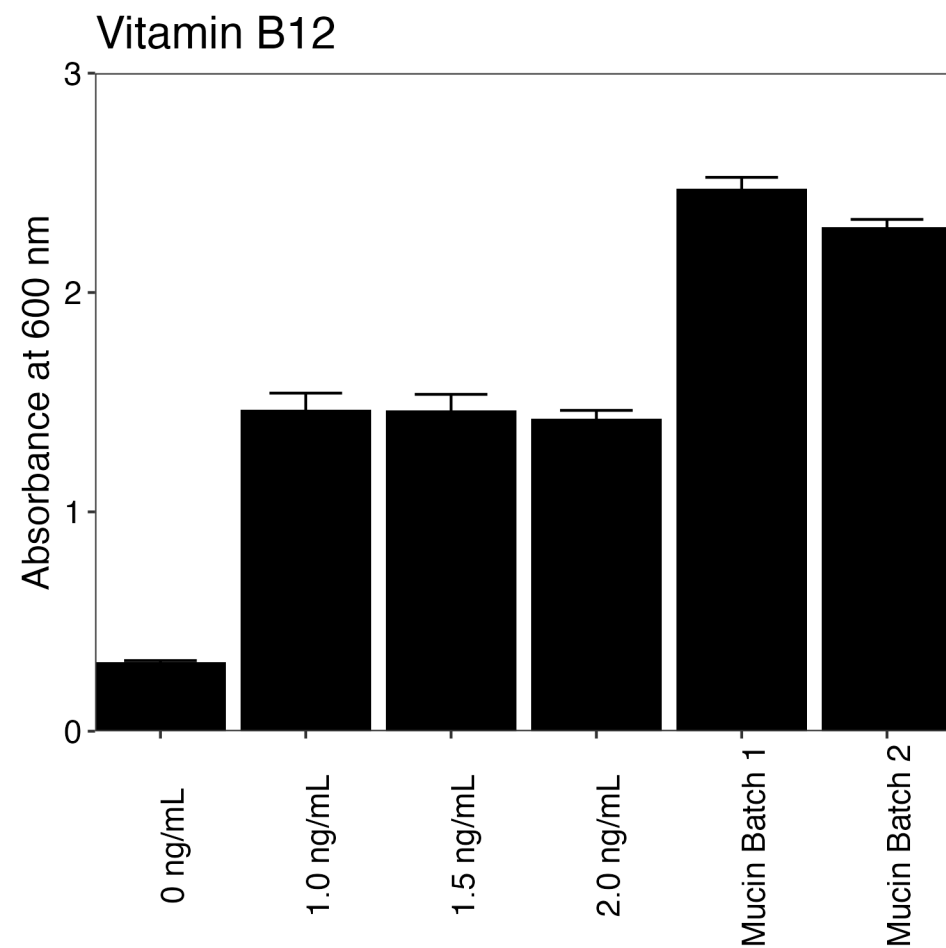

Supplement: Figure S5 — Mucin contains vitamin B12, supporting the growth of Lactobacillus leichmanii. [file spectrum.02071-25-s0009.pdf]
